# Moxibustion combined with chemotherapy inhibits gastric cancer growth by modulating the immunosuppressive microenvironment involving the Treg/IL-10/TGF-β1 axis

**DOI:** 10.3389/fphar.2025.1688182

**Published:** 2025-12-08

**Authors:** Yong Wu, Li Ma, Mengying Mao, Yuling Leng, Xianyang Zhou, Heng Xu, Yanqi Ding, Wanrong Hao, Wanhui Dong, Yonglei Zeng

**Affiliations:** 1 Department of Medical Oncology, The Second Affiliated Hospital of Anhui University of Traditional Chinese Medicine, Hefei, China; 2 Department of Medical Oncology, Guangde Hospital of Traditional Chinese Medicine, Guang De, Anhui, China; 3 Department of Medical Oncology, Lu’an Hospital Affiliated To Anhui University of Chinese Medicine, Lu’an, China

**Keywords:** consolidating the root and nourishing the essence, moxibustion, gastric tumor in mice, regulatory T cells, cytokines

## Abstract

**Background:**

The immunosuppressive microenvironment poses a major challenge in gastric cancer (GC) therapy. Moxibustion, based on the “Guben Peiyuan” theory, shows potential in oncology, but its immunomodulatory mechanisms in GC remain elusive.

**Methods:**

This study integrated bioinformatics analysis with animal experiments. We analyzed pan-cancer expression and prognostic value of IL-10 and TGF-β1 via TCGA/GTEx databases. A mouse model of MFC gastric cancer was established to evaluate the effects of moxibustion (ST36, CV12, CV6, CV4), chemotherapy (5-FU), and their combination on tumor growth and the immune microenvironment.

**Results:**

Bioinformatics indicated that IL-10 and TGF-β1 were upregulated in GC, positively correlated with FOXP3^+^ Treg infiltration and poor prognosis. *In vivo*, the moxibustion + chemotherapy combination demonstrated the most potent tumor inhibition (inhibition rate: 45.9%). Mechanistically, the combination therapy exerted a “dual immunomodulatory effect” on the tumor immune microenvironment. It simultaneously suppressed immunosuppressive components, evidenced by reduced peripheral Tregs (7.02% vs. 3.91%), serum IL-10 (127.21 vs. 51.42 pg/mL) and TGF-β1 (547.84 vs. 266.82 pg/mL) levels, and downregulated Foxp3/TGF-β1 protein in tumors. Concurrently, it enhanced anti-tumor immunity, as evidenced by a significant increase in cytotoxic CD8^+^ T cell infiltration compared to the model group. Notably, the combined therapy elicited the most potent bidirectional immunomodulatory effect: it most effectively suppressed immunosuppressive components (reducing Tregs to 3.91% and serum TGF-β1 to 266.82 pg/mL) while simultaneously maintaining a robust CD8^+^ T cell response (22.8%), thereby achieving optimal overall remodeling of the tumor immune landscape.

**Conclusion:**

This study is the first to demonstrate that moxibustion synergizes with chemotherapy to inhibit gastric cancer growth through bidirectional remodeling of the immune microenvironment, simultaneously attenuating immunosuppression and boosting immune attack. Our findings provide a novel mechanistic insight into the integrated “Guben Peiyuan” and Western medicine strategy for GC.

## 1 Introduction

Gastric cancer is the fifth most common malignant tumor worldwide, with over one million new cases in 2022 and a persistently high mortality rate (Sung et al., 2021). Despite certain advancements in surgery, chemotherapy, and targeted immunotherapy in recent years, patients with advanced gastric cancer still face severe challenges such as treatment resistance, significant side effects, and low survival rates (Ajani et al., 2022). Studies show that CD4^+^CD25+Foxp3+ regulatory T cells (Tregs) are crucial in facilitating tumor immune evasion. These cells suppress the function of effector T cells, promoting tumor immune tolerance (Sakaguchi et al., 2020). In gastric cancer patients, elevated Treg cell levels in peripheral blood and tumor tissues are strongly linked to disease progression and poor prognosis (Geng et al., 2015). Immunosuppressive cytokines like transforming growth factor-β1 (TGF-β1) and interleukin-10 (IL-10) contribute to the enhanced immunosuppressive condition of the tumor microenvironment (Derynck et al., 2021; Mirlekar, 2022). These findings suggest that targeting Treg cells and related immunosuppressive factors may offer a new direction for improving gastric cancer treatment.

Based on the theory of “guben peiyuan” in traditional Chinese medicine (i.e., enhancing the body’s disease resistance by supporting the body’s vital energy), moxibustion, as an important external treatment method of this theory, has been applied in the adjuvant treatment of tumors. Previous studies have shown that moxibustion can enhance the efficacy of chemotherapy and reduce its side effects by regulating the balance of T cell subsets, reducing the levels of inflammatory factors, and improving immune function (Shen et al., 2025). For instance, in lung cancer and breast cancer models, moxibustion combined with chemotherapy can inhibit tumor growth, promote NK cell anti-tumor immunity, improve the tumor immune microenvironment (such as increasing M1 macrophage and Th1 cell infiltration), and alleviate chemotherapy-induced bone marrow suppression and liver toxicity (Wang et al., 2020; Zhang et al., 2025). However, these studies have obvious limitations: most of them focus on lung cancer, breast cancer or liver cancer, lacking direct evidence for gastric cancer models; the depth of mechanism exploration is insufficient. Although changes in the proportion of immune cells have been observed, the molecular pathways regulating the differentiation and function of Treg cells (such as the TGF-β/Smad or IL-10/STAT3 signaling axis) have not been deeply analyzed; most studies on the synergy of combined therapy focus on symptom relief and toxicity reduction rather than the systematic reversal of the immunosuppressive microenvironment; and the selected acupoints and moxibustion parameters vary greatly among different studies, making it difficult to standardize the assessment of the biological basis of the “guben peiyuan” theory.

**FIGURE 1:**
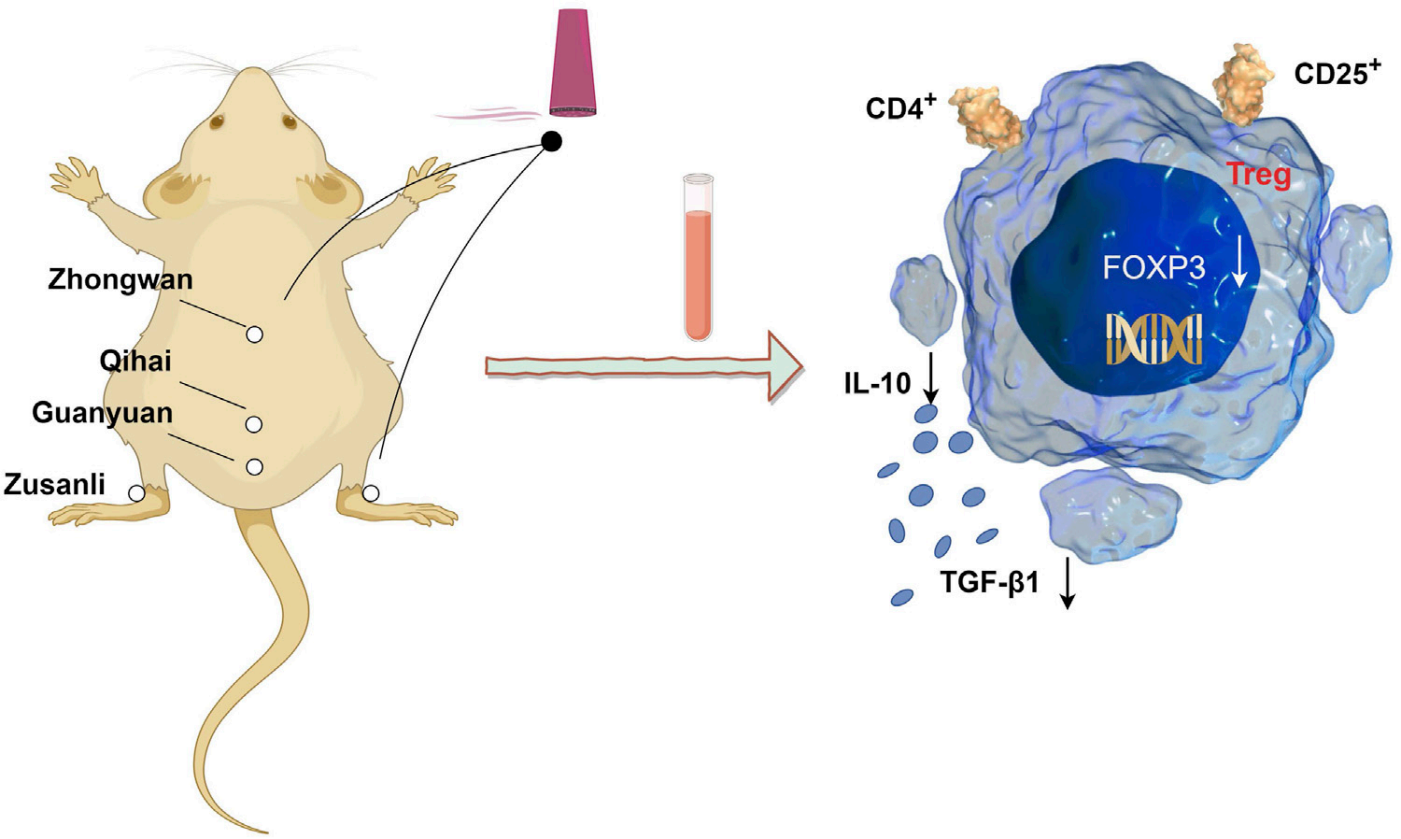
Mechanism diagram of moxibustion intervention in gastric cancer mice for immune regulation and anti-tumor effects. Moxibustion can reverse tumor immune escape and enhance the immune function of the body by regulating and reducing the proportion of CD4^+^CD25+Foxp3+Treg cells in the blood and the expression levels of IL-10 and TGF-β1. The acupoints for the corresponding experimental mice are Zhongwan (CV12), Qihai (CV6), Guanyuan (CV4), and Zusanli (ST36).

To fill this gap, this study innovatively established a mouse model of gastric cancer and, based on the theory of “guben peiyuan”, selected corresponding acupoints for moxibustion intervention: Zhongwan (Li et al., 2021), the gathering point of the stomach, is mainly used to treat diseases of the stomach; Zusanli (Huang et al., 2022), a key point for strengthening the body, has been confirmed by modern research to regulate the immune system; Qihai and Guanyuan (Zhang et al., 2020) are essential points for nourishing the essence and consolidating the root. The combination of these four points embodies the therapeutic principle of “guben peiyuan”. By integrating bioinformatics analysis and animal experiments, this study focused on clarifying the specific inhibitory effect of moxibustion on regulatory T cells (Treg cells) and its regulatory mechanism on the transforming growth factor β1 (TGF-β1)/interleukin 10 (IL-10) pathway, and evaluated its synergistic anti-tumor effect when combined with chemotherapy (Figure 1). This study not only provides empirical evidence for the regulation of tumor immunity by moxibustion in the field of gastric cancer but also lays a theoretical foundation for the deep integration of traditional medicine and modern oncology.

## 2 Materials and methods

### 2.1 Expejrimental medication

The moxa sticks were purchased from Yueyang Aijiantang Biotechnology Co., Ltd. In Hunan Province. They were of the same batch and had specifications of 9 mm*120mm and 4 mm*120 mm.The School of Pharmacy at Anhui University of Chinese Medicine confirmed these as processed Artemisia argyi products from the Compositae family, suitable for the experiment’s requirements.5-Fu was obtained from the Western Pharmacy at the Second Affiliated Hospital of Anhui University of Chinese Medicine, manufactured by Shanghai Xudonghai Pharmaceutical Co., Ltd. (National Medical Product Approval No. H31020593).

### 2.2 Main reagents and instruments

Reagents: The reagents used included CD3-APC, CD4-FITC, CD8-PE, CD25-APC, and FOXP3-PE, all acquired from BioLegend (Beijing) Biotechnology Co., Ltd. Phosphate buffered saline (PBS) was obtained from Shanghai Lianshuo Biotechnology Co., Ltd., and mouse interleukin-10 (IL-10) and mouse transforming growth factor β1 (TGF-β1) were sourced from Wuhan Genmei Technology Co., Ltd. Flow cytometer (Beckman Biotechnology Co., Ltd.), pipette (Dalong Medical Equipment Co., Ltd.), centrifuge (80–2, Shanghai Hexin Science and Education Equipment Co., Ltd.), microplate reader (RT-6100, Lede), vortex mixer (GL-88B, Qilinbei Instrument Manufacturing Co., Ltd.), pipettes, multi-channel pipettes (Eppendorf, Eppendorf), 0.5–10 μL, electric constant temperature incubator (DNP-9052BS-II, Shanghai Sanfa).

### 2.3 Experimental animals

The experiment utilized 24 male SPF BALB/c-nude mice (Shen et al., 2022; Cho et al., 2024; Cho et al., 2023; Xie et al., 2025; Shimizu et al., 2021; Jiang et al., 2023), aged 3–6 weeks, each weighing 20 ± 2 g. The animals were acquired from Zhejiang Vital River Laboratory Animal Technology Co., Ltd., under license number SCXK (Zhe) 2019–0001. MFC mouse gastric cancer cells were obtained from Cyagen Biosciences (Shanghai) Co., Ltd., with the product number iCell-m035. The experiments were conducted at the Key Laboratory of Anhui Province for New Medicine, Anhui University of Chinese Medicine. During the experiment, all mice were kept in the same animal room, fed with the same type of feed and water, and provided with the same type of bedding to maintain a constant and suitable temperature and humidity. They were acclimatized for 1 week. The Laboratory Animal Ethics Committee of Anhui University of Chinese Medicine approved this project (Approval No. AHUCM-mouse-2023127).

### 2.4 Establishment of gastric tumor model in mice

MFC mouse anterior gastric cancer cells were cultured in a medium composed of 90% culture medium, 10% fetal bovine serum, and 1% antibiotics. The samples were incubated at 37 °C with 5% CO2, and the medium was refreshed bi-daily. When the cells covered about 90% of the culture dish, they were subcultured at a ratio of 1:3 into new culture dishes and placed back in the incubator. MFC mouse anterior gastric cancer cells were collected and prepared into a 1 × 10^7 cells/mL suspension under sterile conditions. The mice’s right axilla was disinfected with iodophor, and 0.2 mL of cell suspension was administered into each mouse’s right axilla using a 1 mL syringe. Daily observations were conducted on the mice, and the experiment proceeded to the next phase once the tumor reached a 3 mm diameter, indicating successful modeling. Following successful modeling, 24 mice were randomly assigned to four groups of six (Mao et al., 2023; GUO Lu et al., 2022): the model group, moxibustion group, chemotherapy group (5-FU group), and moxibustion combined with chemotherapy group (moxibustion + 5-FU group).

### 2.5 The treatment of different groups

Moxibustion Group (Pan et al., 2021): The acupoint location was based on the “Experimental Acupuncture” edited by Guo Yi (Guoyi, 2016) (a textbook of the 14th Five-Year Plan of China Traditional Chinese Medicine Publishing House). Mild heat moxibustion was applied to the acupoints Zusanli (ST 36), Zhongwan (CV 12), Qihai (CV 6), and Guanyuan (CV 4). Mild moxibustion was administered bilaterally at Zusanli, along with the abdominal acupoints Zhongwan, Qihai, and Guanyuan, using ignited moxa sticks. A fine moxa stick with a diameter of 4 mm was used for the bilateral Zusanli points, held approximately 1–2 cm above the skin to ensure gentle thermal stimulation. Due to the close anatomical positioning of Zhongwan, Qihai, and Guanyuan on the mouse abdomen, a broader moxa stick with a 9 mm diameter was utilized at these sites, maintaining the same distance from the skin surface. Each acupoint received treatment for 15 min per session, conducted once daily over a 2-week period. To ensure the consistency of the intensity of heat stimulation, the moxa stick was fixed 1–2 cm above the acupoint by the same operator during moxibustion. The stimulation intensity was standardized using a dual-criteria approach. The appearance of a slight flush (Li haiyan et al., 2023; Ouyang et al., 2022) on the local skin was employed as the endpoint for biological efficacy, while the skin surface temperature was maintained below 39 °C as the objective safety parameter (Datta et al., 2015; MacArthur Clark and Sun, 2020). During the procedure, mice were restrained but did not receive any injections.

Chemotherapy Group: Mice were administered fluorouracil intraperitoneally at a dose of 20 mg/kg (Fu et al., 2018). Before injection, the lower abdominal area was sterilized with 75% ethanol. Using a 1 mL syringe, the drug was delivered into the peritoneal cavity at an angle of about 45° to minimize the risk of damaging internal organs or blood vessels, with slow injection to ensure proper dispersion. The treatment schedule involved administration every other day—specifically on days 1, 3, 5, 7, 9, 11, and 13—resulting in seven doses throughout the 14-day experimental period.

Combined Moxibustion and Chemotherapy Group: This group underwent both moxibustion and chemotherapy treatments simultaneously. The moxibustion procedure mirrored that of the Moxibustion Group, with daily application at the specified acupoints. The chemotherapy protocol was identical to that used in the Chemotherapy Group. To simulate real-world combination therapy and reduce handling stress, moxibustion was carried out at a consistent time each day, followed by fluorouracil injection 1 hour later on the same days as chemotherapy administration.

Model Group: After successful model induction, these mice were maintained under the same standard housing conditions as the other groups. At the times corresponding to chemotherapy administration, they received intraperitoneal injections of an equal volume of normal saline. During the scheduled moxibustion sessions, they were subjected to the same duration of restraint without undergoing actual moxibustion or receiving any drug treatment.

### 2.6 Bioinformatics methods

#### 2.6.1 Data sources and preprocessing

RNA-Seq raw count matrices and clinical data for 33 solid tumor types from TCGA (The Cancer Genome Atlas) were sourced from the GDC Data Portal (https://portal.gdc.cancer.gov), using STAR-Counts and GRCh38. Expression data for GTEx normal tissues (adjacent non-tumor tissues) were retrieved from the GTEx official website (https://gtexportal.org/home/datasets, V8 version). All data were uniformly normalized by log2 (TPM +1). A total of 10,228 samples, each with RNAseq data and clinical information, were retained for analysis. The clinical data encompassed overall survival (OS) from the TCGA Pan-Cancer Clinical Data Resource (TCGA-CDR). Kaplan -Meier curves were analyzed using logrank tests and univariate Cox regression to obtain p-values and hazard ratios (HR) with 95% confidence intervals (CI).

#### 2.6.2 Pan-cancer differential expression analysis

Differential expression analysis on TCGA tumor tissues and GTEx normal tissues was performed using DESeq2 in R software version 4.0.3. Differential expression results for IL-10 and TGF-β1 across all cancer types were extracted and visualized using ggplot2 box plots.

#### 2.6.3 The correlation between gene co-expression and immune cell infiltration

In the TCGA-STAD gastric cancer cohort (n = 375), Spearman rank correlation analysis assessed the relationships among Foxp3, IL-10, and TGF-β1 mRNA expressions, calculating the correlation coefficient (r) and P value. The TIMER2.0′Immune Association’ module (http://timer.cistrome.org) was employed to assess the correlation between FOXP3 expression and the abundance of tumor-infiltrating CD4^+^CD25+Foxp3+ Tregs, as inferred by the EPIC algorithm. The significance threshold was established at a false discovery rate (FDR) of less than 0.05.

#### 2.6.4 The distribution of immune checkpoint gene expression

The genes ITPRIPL1, SIGLEC15, TIGIT, CD274, HAVCR2, PDCD1, CTLA4, LAG3, and PDCD1LG2 play crucial roles in immune regulation. Examining gene expression levels enhances our understanding of immune checkpoint functionality. We analyzed STAR-counts data from 375 gastric cancer cases, incorporating clinical information, and applied log2 (TPM +1) normalization to the extracted TPM-formatted data. For subsequent analysis, we utilized 188 samples with high IL-10 and TGFβ1 expression, 187 samples with low expression of these markers, and 391 normal tissue samples, all sourced from the TCGA and GTEx databases, after filtering for RNAseq data and clinical information.

#### 2.6.5 Survival and prognostic analysis

The Kaplan-Meier Plotter gastric cancer module assessed the relationship between IL-10 and TGF-β1 expression levels and patient overall survival (OS). The ggrisk package was utilized to visualize the association between gene expression and survival status. Using the surv_cutpoint function from the survminer package, the optimal cut-off value was determined, categorizing the samples into high-expression and low-expression groups. Survival differences were assessed with the log-rank test, and the ROC curve was generated using the timeROC package.

#### 2.6.6 Single-cell transcriptome validation

The single-cell RNA-seq dataset GSE163558 for gastric cancer (10× Genomics) was obtained from the GEO database. Data quality control was performed using Seurat v4.3.0 (filtering criteria: genes detected in less than three cells and cells with less than 200 detected genes). Cells with fewer than 200 or more than 2500 detected genes, and those with mitochondrial content exceeding 5%, were excluded. After discarding the poor-quality cells, we used scale. Factor = 10,000 to scale the UMI counts for each cell to standardize the data. After logarithmic transformation of the data, we used the ScaleData function of Seurat (v4.1.0). The corrected normalized data was used for standard analysis, and the top 2000 variable genes were extracted for principal component analysis (PCA). For t-SNE visualization and clustering, we used the ElbowPlot function to evaluate the principal components and retained the top 20 principal components. Cell clustering was conducted with the Seurat R package’s FindClusters function, using a resolution of 0.8. Cell annotation utilized the MAESTRO and SingleR packages in R, referencing datasets such as HumanPrimaryCellAtlasData, BlueprintEncodeData, DatabaseImmuneCellExpressionData, MonacoImmuneData, and NovershternHematopoieticData. Finally, we extracted Tregs and immune-related high-expression subgroups (FOXP3, IL10, TGF-β1, CTLA4), and analyzed their differential expression between the tumor group and the normal group through t-SNE dimensionality reduction visualization.

### 2.7 Observation indicators and measurement methods for mice

During the experiment, the body weight of the mice was recorded daily. From the sixth day after inoculation, the longest diameter (L) and the maximum transverse diameter (W) of the tumor were measured every 3 days using a vernier caliper, and the tumor volume was calculated according to formula V = (L × W^2^)/2.

After the intervention, the mice were sacrificed by cervical dislocation, and blood samples were immediately collected from the eyeballs and spleens. The tumor was completely dissected and weighed, and the tumor mass was accurately recorded. The tumor inhibition rate was calculated according to the following formula: Tumor inhibition rate (%) = [(average tumor mass of the model group - average tumor mass of the treatment group)/average tumor mass of the model group] × 100%. At the same time, tumor tissues were collected: after weighing the tumor, about 3 mm^3^ of tissue blocks were taken and immediately immersed in 10% neutral buffered formalin for fixation, which was used for subsequent paraffin embedding, sectioning and HE staining. The remaining tumor tissues were quickly placed in a −80 °C ultra-low temperature refrigerator for storage, which was used for subsequent protein extraction and Western blot analysis.

#### 2.7.1 HE staining

After the tissue was fixed for 7 h, it was rinsed with running water for 6 h. The automatic dehydrator program was set to perform dehydration and clearing. The tissue was immersed in wax, and the paraffin block was placed on the cooling machine for 30 min to cool. Then, 3–4 μm thin sections were cut, and qualified thin sections were placed on the slides. The slides were baked overnight at 65 °C in an electric constant temperature box. The sections were deparaffinized by sequential immersion in xylene I, II, and III for 10 min each, followed by placement in gradient alcohol for 5 min per step. The sections were washed, stained with hematoxylin for 2 min, differentiated in 1% hydrochloric acid alcohol for 5 s, and then rinsed. The sections were stained for 2 min using a 0.5% eosin aqueous solution. The sections underwent a reverse deparaffinization process by being immersed in gradient alcohol for 5 min each, followed by clearing in xylene I, II, and III for 30 min each. After adding neutral gum, the coverslips were placed to seal the sections. The sections were examined under a microscope post-sealing.

Specimen observation: To ensure the objectivity and representativeness of the assessment, two pathologists who were unaware of the group information selected five non-overlapping fields (×200 magnification) from the center and edge regions of each slice using systematic random sampling for observation and image acquisition.

Criteria for necrosis identification: The presence of necrosis was identified based on internationally recognized morphological features of cell death (Galluzzi et al., 2018). These features include areas where cells exhibit nuclear pyknosis (nuclear condensation and deep staining), nuclear fragmentation (nuclear membrane rupture and chromatin fragmentation), or nuclear dissolution (complete disappearance of the nucleus), accompanied by enhanced eosinophilic staining of the cytoplasm and loss of cellular structure.

Grading of necrotic area extent: Following a pre-established semi-quantitative scoring system (Ye et al., 2023), the percentage of necrotic area in the entire tumor slice was evaluated under low magnification (×5 objective lens), and the following grades were assigned:

- 0 points (none/minimal): No necrosis or necrotic area <10%
- 1 point (moderate): Necrotic area 10%–30%
- 2 points (significant): Necrotic area ≥30%

Grading of tumor cell density: Tumor cell density was classified using a four-level semi-quantitative method (Bor et al., 2019): 0 points: no cells or only a few tumor cells; 1 point: less than 2 malignant cell clusters with at least 10 tumor cells; 2 points: 2 to 4 malignant cell clusters with at least 10 tumor cells; three points: more than 4 malignant cell clusters with at least 10 tumor cells.

Inter-rater consistency test: To ensure the objectivity and reproducibility of the scoring, two raters independently scored 30% of the randomly selected samples (n = 8). Kappa statistics were used to test the consistency between raters, and the results showed good consistency (Kappa >0.8). The remaining samples were scored by one of the main raters.

#### 2.7.2 Flow cytometry analysis of T cell subsets and treg cells

Peripheral blood sample preparation: Following blood collection from the mouse orbital sinus and spleen, anticoagulation was achieved using sodium heparin. To enrich the lymphocyte fraction and enhance the accuracy of flow cytometric analysis, peripheral blood mononuclear cells (PBMCs) were separated from whole blood via density gradient centrifugation employing Ficoll solution, followed by cell staining.

- Analysis of T cell subsets (CD3^+^CD4^+^ and CD3^+^CD8^+^): Around 1 × 10^6^ PBMCs were aliquoted and incubated with fluorochrome-conjugated antibodies specific for CD3-APC (BioLegend, cat. 201412, lot B292851), CD4-FITC (BioLegend, cat. 201505, lot B374477), and CD8-PE (BioLegend, cat. 200610, lot B316147). After thorough mixing by vortexing, the samples were protected from light and incubated at room temperature for 15 min. Cells were then washed twice with phosphate-buffered saline (PBS, Hyclone, cat. SH30256-01), resuspended in buffer, and analyzed on a Beckman CytoFLEX flow cytometer. FlowJo V10 software was used for subsequent data interpretation.
- Quantification of regulatory T cells (Treg; CD4^+^CD25^+^FOXP3^+^): Approximately 1 × 10^6^ PBMCs were stained with CD4-FITC (BioLegend, cat. 201505, lot B336282) and CD25-APC (BioLegend, cat. 202113, lot B371435) for surface marker labeling. The mixtures were gently vortexed and incubated in the dark at room temperature for 15 min. After a single PBS wash, cells were fixed and permeabilized using the True-Nuclear™ Transcription Factor Buffer Set (BioLegend) according to the manufacturer’s protocol. Intracellular staining was performed by adding FOXP3-PE antibody (BioLegend, cat. 320007, lot B346123), followed by a 30-min dark incubation at room temperature. Post-staining, cells were washed, resuspended, and analyzed via flow cytometry (Beckman CytoFLEX). Data processing was carried out using FlowJo V10.

#### 2.7.3 Enzyme-linked immunosorbent assay (ELISA)

After the mice were sacrificed, blood was collected and left at room temperature for 10–20 min. Then, it was centrifuged at 2000–3,000 rpm for 20 min. The upper serum was carefully collected, aliquoted and stored at −80 °C for future analysis. The concentrations of interleukin-10 (IL-10) and transforming growth factor-β1 (TGF-β1) in the serum were determined using commercial mouse ELISA kits (GeneMed Biotech Co., Ltd., Wuhan, China) following the manufacturer’s instructions. The specific detection parameters for the two indicators are as follows:

- IL-10 detection: The mouse IL-10 ELISA kit (cat. JYM0005Mo) was used. The principle of the experiment is the double antibody sandwich method. The steps are as follows: Set up standard wells and sample wells on the microplate coated with anti-mouse IL-10 antibody. Dilute the standard in a series and add it to the wells in sequence to generate a standard curve. The final concentration gradient of the standard is 90, 60, 30, 15, and 7.5 pg/mL. Dilute the serum samples to be tested with sample diluent at a ratio of 1:5 and add 50 μL to each well. Seal the plate with a sealing film and incubate at 37 °C for 30 min. Wash the plate 5 times, then add 50 μL of HRP-labeled detection antibody to each well and incubate at 37 °C for another 30 min. Wash the plate 5 times, then add 50 μL of chromogen A and 50 μL of chromogen B to each well in sequence, and incubate at 37 °C in the dark for 10 min. Finally, add 50 μL of stop solution to each well to terminate the reaction, and immediately measure the absorbance (OD value) of each well at a wavelength of 450 nm. Calculate the concentration of IL-10 in the samples based on the standard curve.
- TGF-β1 detection: The mouse TGF-β1 ELISA kit (cat. JYM0215Mo) was used. The operation steps, incubation and color development time, detection wavelength, and sample volume were exactly the same as those of the IL-10 detection kit, but the concentration gradient of the standard was different, being 180, 120, 60, 30, and 15 pg/mL. Calculate the concentration of TGF-β1 in the samples based on its specific standard curve.

#### 2.7.4 Western blotting detection

The six mice in each group were randomly divided into three pairs. For each pair, equal amounts of tumor tissue from the two mice were combined to form one independent pooled sample (Suzuki et al., 2011). For each mixed tissue sample (approximately 100 mg), add 600 μL of RIPA cell lysis buffer (Beyotime, catalog number P0013B, batch number 09271919023, containing 0.6 mM PMSF), and homogenize thoroughly on ice. The homogenate was centrifuged at 12,000 × g for 15 min at 4 °C, and the supernatant was collected as the total protein. An appropriate amount of the protein supernatant was mixed with 5× SDS-PAGE protein loading buffer at a volume ratio of 1:4 and heated at 100 °C for 10–15 min to fully denature the proteins.

SDS-PAGE gel electrophoresis was performed using 10% resolving gel, with 40 μg of total protein loaded per well. Electrophoresis was carried out at a constant voltage of 80 V for approximately 1 h. After electrophoresis, the proteins were transferred to a PVDF membrane (Millipore, lot: R7SA9081E) using the wet transfer method. Before transfer, the PVDF membrane was activated by soaking in methanol for 2–3 min. The transfer was conducted under constant current conditions, and the transfer time was set according to the molecular weight of the target protein: Foxp3 (47 kDa) for 50 min, TGF-β1 (55 kDa) for 55 min, and GAPDH (36 kDa) for 40 min.

After transfer, the membrane was blocked with 5% skim milk in PBST at room temperature for 2 h. After blocking, the membrane was incubated with the specific primary antibodies at 4 °C overnight. The primary antibodies and their dilution ratios were as follows: rabbit polyclonal antibody against Foxp3 (Bioss, cat. Bs-23074R, lot: AF03154485), diluted 1:1,000; rabbit polyclonal antibody against TGF-β1 (Bioss, cat. Bs-0086R, lot: AG19301,531), diluted 1:2000; mouse monoclonal antibody against GAPDH (Zhongshan Jinqiao, cat. TA-08, lot: 230040220), diluted 1:2000.

After overnight incubation with the primary antibodies, the membrane was washed three times with PBST for 10 min each. Then, it was incubated with HRP-labeled secondary antibodies at room temperature for 1.2 h. The secondary antibodies and their dilution ratios were as follows: HRP-labeled goat anti-rabbit IgG (Zhongshan Jinqiao, lot: 202700514), diluted 1:20,000; HRP-labeled goat anti-mouse IgG (Zhongshan Jinqiao, lot: 140,193), diluted 1:20,000. After incubation with the secondary antibodies, the membrane was washed three times with PBST for 10 min each.

Finally, chemiluminescence was performed using an ECL ultra-sensitive luminescence kit (Thermo, lot: VC298015). The gray values of the target bands were quantitatively analyzed using ImageJ software (National Institutes of Health). GAPDH was used as the internal reference, and the relative expression levels of the target proteins were calculated as the ratio of their gray values to the corresponding GAPDH gray values. This process generated three independent biological replicates per group (n = 3). Data are presented as mean ± standard deviation and analyzed statistically.

### 2.7 Statistical methods

Data from the animal experiment (four groups, n = 6 per group) were analyzed using SPSS Statistics 26.0 (IBM Corp.). The sample size is consistent with established practices in preclinical studies (Jin et al., 2024). The normality of data distribution and homogeneity of variances were assessed for each dataset using the Shapiro-Wilk test and Levene’s test, respectively. This step ensured the correct selection of subsequent parametric or non-parametric tests. For multiple group comparisons, one-way ANOVA followed by the Student-Newman-Keuls (SNK) *post hoc* test was applied to data meeting both assumptions of normality and homogeneity of variance. Otherwise, the non-parametric Kruskal–Wallis H test was employed, followed by the Nemenyi test for *post hoc* comparisons. Longitudinal data (body weight and tumor volume) were analyzed using repeated measures ANOVA. If a significant interaction between time and group was observed, simple effect analyses with Bonferroni correction for multiple time points were planned. For semi-quantitative pathological scores, the Kruskal–Wallis H test was used. Given the four groups necessitated six pairwise comparisons per indicator, the Nemenyi *post hoc* test was conducted with a Bonferroni-adjusted significance level (α' = 0.05/6 ≈ 0.0083).

Bioinformatics analyses were conducted in the R environment (version 4.0.3). Pan-cancer differential expression analysis was performed using DESeq2, with genes having an FDR-adjusted p-value <0.05 considered significant. In the TCGA-STAD cohort, Spearman’s rank correlation was used to assess relationships among FOXP3, IL-10, and TGF-β1 mRNA levels. Survival analysis was performed using the Kaplan-Meier method with the log-rank test, and the Cox proportional hazards model was used to calculate hazard ratios (HR) and 95% confidence intervals. Single-cell RNA-seq data (GSE163558) were processed with Seurat (v4.3.0). Cells were filtered if they expressed <200 or >2500 genes, or if mitochondrial counts exceeded 5%. Data were normalized (scale factor = 10,000), log-transformed, and scaled. Dimensionality reduction utilized PCA on the top 2000 variable genes, with the top 20 principal components selected for graph-based clustering (FindClusters, resolution = 0.8). Immune infiltration was analyzed via the EPIC algorithm on TIMER2.0.

Statistical significance was set at α = 0.05 (two-sided). Exact p-values are reported, and data are presented as mean ± standard deviation. Data visualization was assisted by GraphPad Prism 8.02, ggplot2, survminer, and timeROC.

## 3 Results

### 3.1 Pan-cancer analysis of IL-10 and TGF-β1 expression levels

#### 3.1.1 The expression heterogeneity between tumors and normal tissues

**FIGURE 2:**
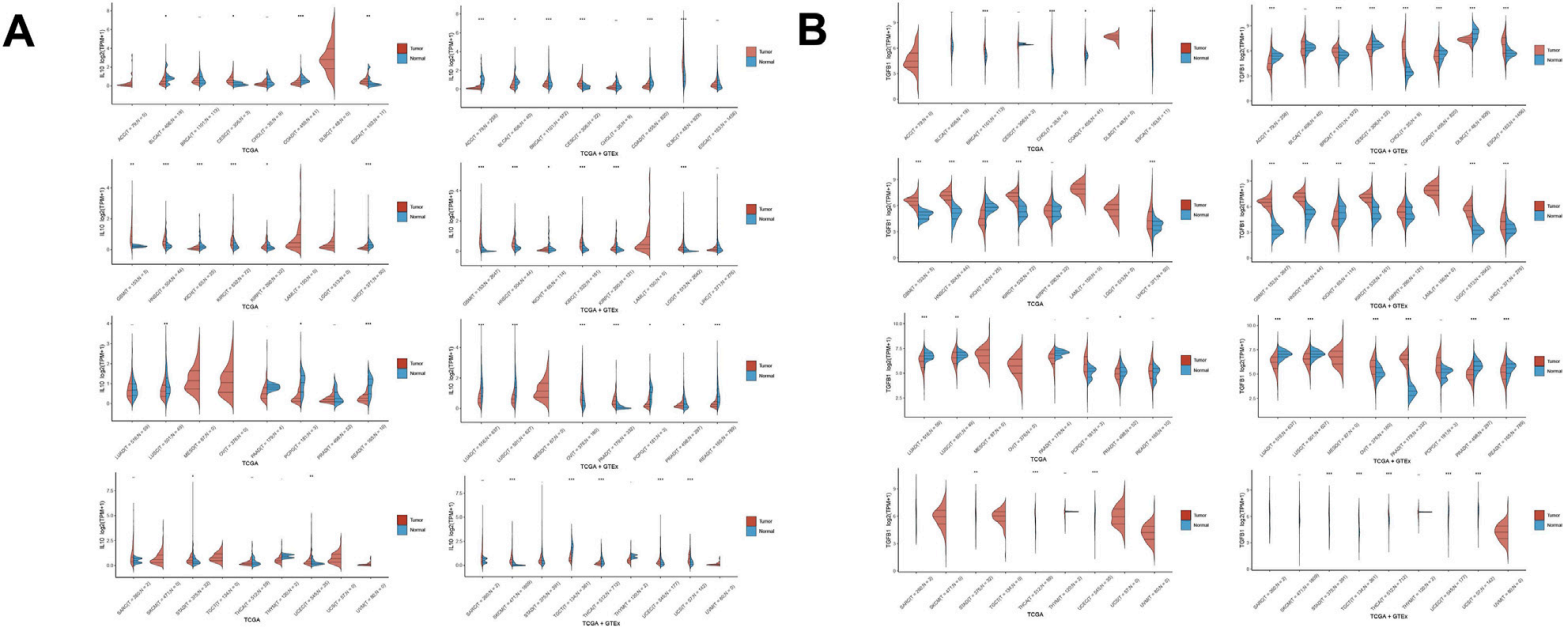
Box plots of the expression distribution of immune-related genes IL-10 **(A)** and TGFβ1** (B)** in tumor tissues and normal tissues of multiple cancer types. The horizontal axis represents different sample groups, and the vertical axis indicates the expression distribution of the gene. Different colors represent different groups, with red representing tumor tissues and blue representing normal tissues. The asterisks in the upper left corner represent the significance p-values, where * indicates a p-value less than 0.05, ** indicates a p-value less than 0.01, and *** indicates a p-value less than 0.001. The significance of the two groups of samples was determined by the Wilcoxon test.

In both the TCGA and TCGA + GTEx integrated cohorts, significant expression differences are observed between tumor (red) and normal (blue) tissues across most cancer types. Taking the IL-10 family molecules in Figure 2A as an example, the violin plots of different cancer types (such as BRCA, LIHC, etc.) vary: in some cancer types, the expression distribution of tumor tissues is significantly shifted upwards (for instance, the red area in specific cancer types covers a higher expression range), suggesting upregulation of this factor in tumors; while in a few cancer types, the expression distribution of tumor tissues shifts leftward, with an increased proportion of low-expression intervals, indicating possible downregulation. This difference remains consistent in the TCGA + GTEx cohort, verifying the cancer-type specificity of expression heterogeneity.

In gastric cancer (STAD), analysis of IL-10-related factors reveals that the expression distribution in tumor tissues (red) significantly shifts upward compared to normal tissues (blue) in both the TCGA and TCGA + GTEx cohorts, as demonstrated by the violin plot, indicating a larger proportion of high-expression intervals. This clearly indicates a significant upregulation trend of IL-10 family molecules in gastric cancer, suggesting their possible involvement in the construction of the gastric cancer immune microenvironment. In the analysis of TGF-β1-related factors (Figure 2B), gastric cancer tumor tissues also exhibit a consistent upregulation feature, with the red expression distribution significantly higher than the blue normal tissues, further confirming the abnormal activation of immunosuppressive factors in gastric cancer.

#### 3.1.2 The coordinated differences in multi-factor expression profiles

By comparing Figure 2A (IL-10 related) with Figure 2B (TGF-β1 related), it can be seen that there is a coordinated pattern of differential expression of immunosuppressive factors. In liver (LIHC) and breast cancers (BRCA), both IL-10 and TGF-β1 family molecules show increased expression in tumor tissues, indicating a coordinated activation of the immunosuppressive microenvironment. Conversely, in thyroid cancer (THCA), the expression difference is less pronounced, potentially reflecting a unique immune escape mechanism.

In gastric cancer, IL-10 and TGF-β1 family molecules are significantly and concurrently upregulated in tumor tissues. In gastric cancer tissues, IL-10 and TGF-β1-related factors exhibit overlapping high expression intervals, as shown by the synchronous expansion of high expression regions in the red violin plot. This suggests that these molecules collaboratively exert immunosuppressive effects, shaping a tumor-promoting microenvironment.

#### 3.1.3 Queue stability with expression differences

The results of the TCGA single cohort and the TCGA + GTEx integrated cohort were highly consistent: the expression difference trends (upregulation/downregulation directions) and expression distribution ranges (the widening and narrowing of violin plots) between tumor and normal tissues were basically the same. Taking lung cancer (LUAD, LUSC) as an example, the high expression characteristics of immune factors in tumor tissues were stably presented regardless of whether GTEx normal samples were integrated, proving that the differential expression pattern was not significantly affected by the normal tissue source cohort and enhancing the reliability of the results. In gastric cancer, the expression patterns of IL-10 and TGF-β1 family molecules consistently differed between tumor and normal tissues, as observed in both the TCGA single cohort and the TCGA + GTEx integrated cohort analyses. In gastric cancer tumor tissues, the distribution characteristics of high expression of immunosuppressive factors were continuously presented, with the red violin plots always significantly higher than the blue normal tissues, fully demonstrating that the abnormal expression patterns of immune-related factors in gastric cancer have cohort stability and are inherent molecular phenotypes of the gastric cancer immune microenvironment. In conclusion, the expression of immune suppression-related factors varies between tumor and normal tissues across different cancer types, demonstrating both cancer type specificity and cohort stability. The coordinated regulation of IL-10 and TGF-β1 family molecules offers crucial molecular evidence for understanding tumor immune microenvironment heterogeneity and guiding targeted interventions. In gastric cancer, the notable upregulation of IL-10 and TGF-β1 family molecules plays a crucial role in forming an immunosuppressive microenvironment, offering valuable molecular insights for identifying immunotherapy targets and developing combined intervention strategies.

### 3.2 Correlation analysis among FOXP3, IL-10, and TGF-β1 in gastric cancer

#### 3.2.1 Correlation analysis of immune regulatory molecules

**FIGURE 3:**
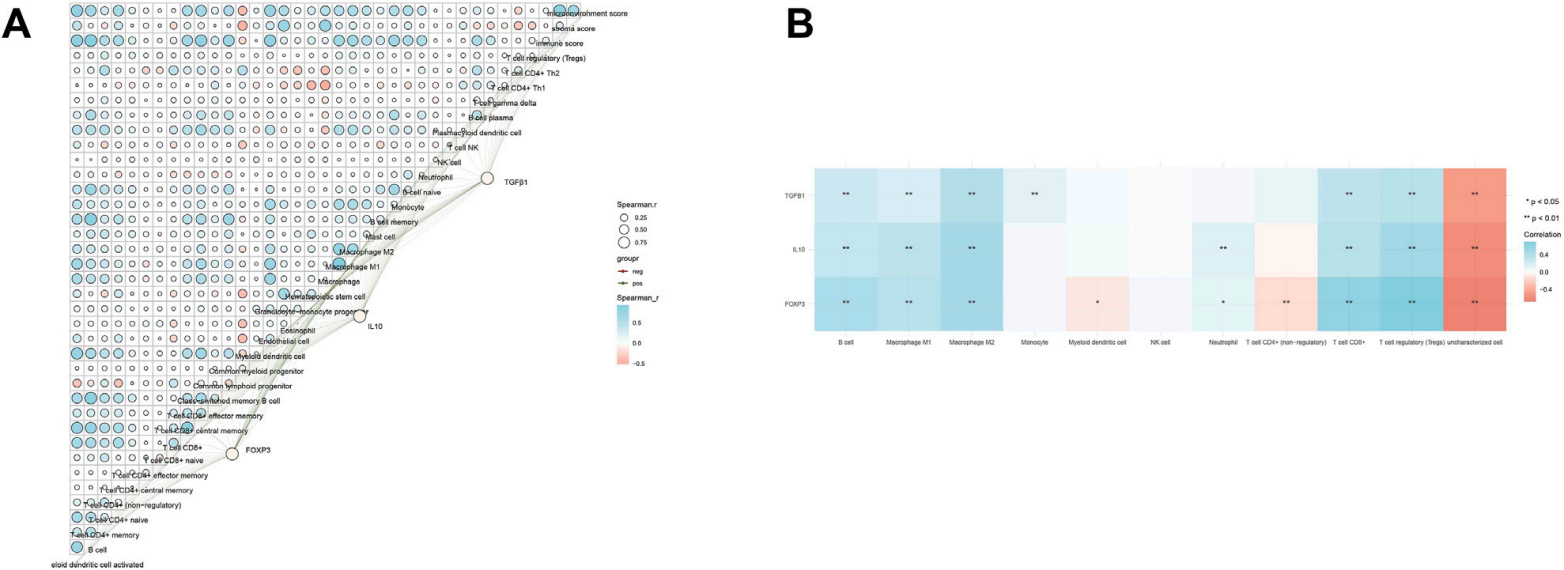
Correlation analysis among FOXP3, IL-10, and TGF-β1 in gastric cancer. In gastric cancer tissues, FOXP3, IL-10 and TGFβ1 are all associated with immune cells to varying degrees **(A)**, and have a strong association with Treg cells **(B)**.

Spearman rank correlation analysis (Figure 3A) revealed a significant synergy among the mRNA expressions of Foxp3, IL-10, and TGF-β1. IL-10 expression showed a positive correlation with TGF-β1 (Spearman r > 0.5), indicating potential transcriptional co-regulation in gastric cancer. Foxp3 showed a positive correlation with IL-10 and TGF-β1 (r ≈ 0.5), suggesting that these immune-suppressive molecules exhibit consistent expression patterns and collectively influence the immune microenvironment in gastric cancer.

#### 3.2.2 The correlation with tumor-infiltrating immune cells

The TIMER2.0′ Immune Association’ module analysis (Figure 3B) indicated a significant correlation between the expression of FOXP3, IL-10, and TGF-β1 and the abundance of tumor-infiltrating CD4^+^ CD25^+^ Foxp3+ Tregs, as inferred by the EPIC algorithm. TGF-β1 and IL-10 showed a strong positive correlation with Treg abundance (r > 0.4, *p* < 0.01), with Foxp3 being the most closely associated core marker of Tregs. The expression of the three factors was positively correlated with the abundance of immune suppressive cells, including macrophages (particularly M2 type) and monocytes (FDR <0.05), while negatively correlated with anti-tumor immune cells like NK cells and cytotoxic T cells. This suggests that Foxp3, IL-10, and TGF-β1 collaboratively create an immune suppressive microenvironment by attracting immune suppressive cells and hindering anti-tumor immune cell infiltration.

In gastric cancer, Foxp3, IL-10, and TGF-β1 create a tightly coordinated immunosuppressive synergistic network. This network not only maintains elevated levels of immune suppressive molecules but also recruits suppressive cells like Tregs and M2 macrophages while inhibiting NK and cytotoxic T cell functions, thereby facilitating an ‘immune escape’ tumor microenvironment through dual mechanisms. This cooperative effect is consistently presented in the Spearman correlation coefficient and the immune cell association heatmap, verifying the core regulatory pattern of the immunosuppressive synergistic network in gastric cancer.

Foxp3, IL-10, and TGF-β1 exhibit significant co-expression in gastric cancer and are strongly associated with the presence of tumor-infiltrating immune cells. By simultaneously enhancing expression, attracting immune suppressive cells, and inhibiting anti-tumor immune responses, they collectively foster an immune suppressive microenvironment. This provides crucial molecular evidence for developing gastric cancer immunotherapy targets, such as combined targeting of Tregs and immune suppressive cell recruitment pathways, and establishes a basis for understanding immune escape mechanisms in gastric cancer.

The correlation between immune checkpoint gene expression and IL-10, TGF-β1 levels in gastric cancer.

**FIGURE 4:**
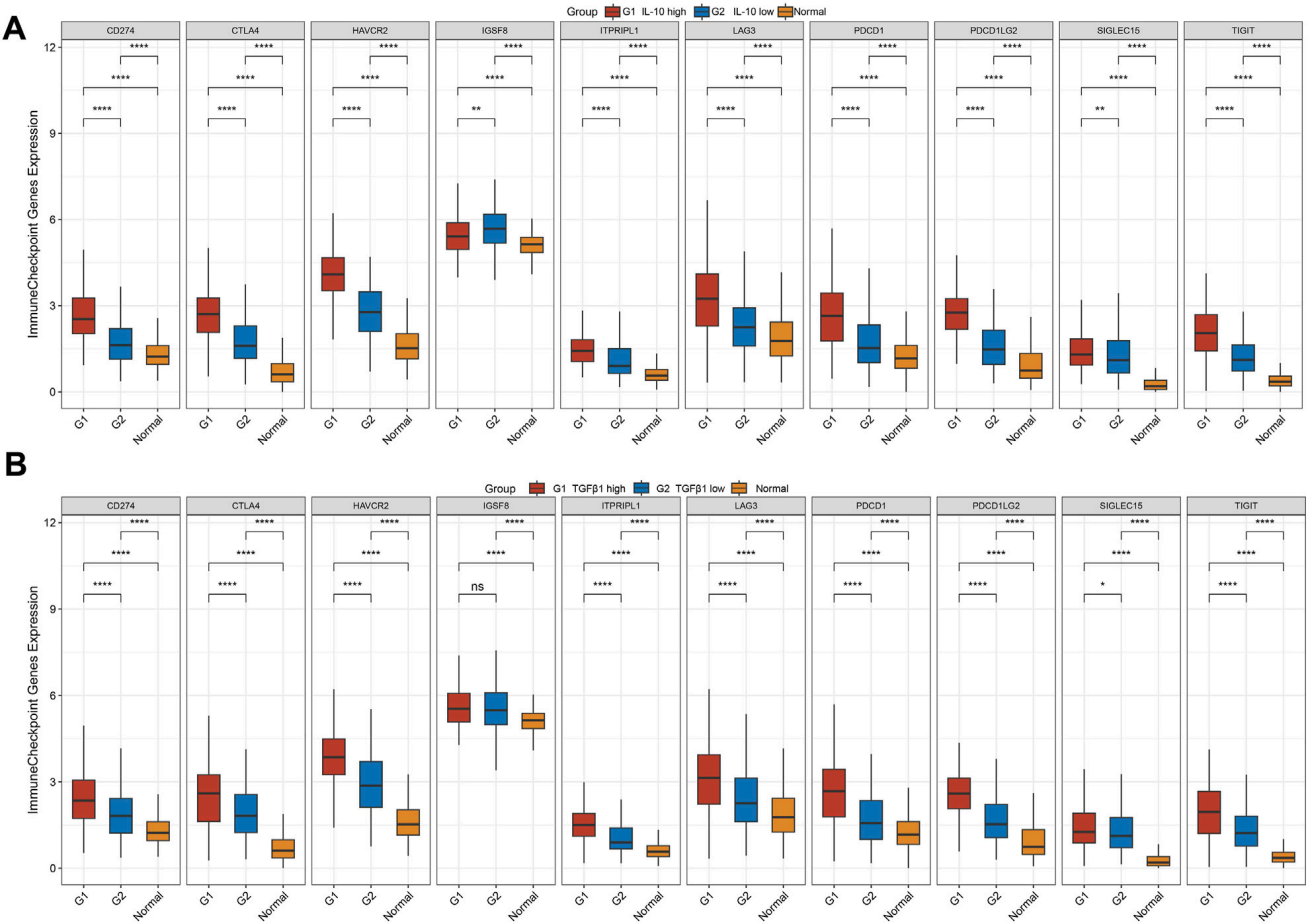
The expression distribution of immune checkpoint genes in tumor tissues and normal tissues. In the figure, the horizontal axis represents different sample groups, and the vertical axis represents the expression distribution of the gene. Red indicates high expression, blue indicates low expression, and yellow indicates the normal group. **(A)** The expression differences of immune checkpoint genes in the IL-10 high-expression group, IL-10 low-expression group, and normal tissues of gastric cancer. **(B)** The expression differences of immune checkpoint genes in the TGFβ1 high-expression group, TGFβ1 low-expression group, and normal tissues of gastric cancer. ns:*p* > 0.05, *p ≤ 0.05, **p ≤ 0.01, ***p ≤ 0.001, ****p ≤ 0.0001.

Figure 4A illustrates that in the high IL-10 expression group (G1), genes including CD274, CTLA4, and HAVCR2 are expressed at significantly higher levels compared to the low expression group (G2) and the normal group (*p* < 0.01), indicating activation of the immune checkpoint pathway with elevated IL-10 expression.

Stratified analysis of TGF-β1 expression (Figure 4B) revealed that the high expression group (G1) exhibited significantly elevated levels of genes like CD274 and CTLA4 compared to G2 and the normal group (*p* < 0.01), suggesting a cooperative upregulation of immune checkpoint molecules with increased TGF-β1 expression.

In groups with elevated IL-10 and TGF-β1 expression, immune checkpoint genes exhibited consistent upregulation, with significantly higher expression in tumor samples compared to normal samples. This confirms the abnormal activation of the immune checkpoint pathway within the tumor microenvironment.

In summary, elevated IL-10 and TGF-β1 levels in gastric cancer synergistically enhance immune checkpoint gene expression, including CD274 and CTLA4, thereby strengthening the immunosuppressive microenvironment and informing immune therapy resistance mechanisms and combined targeted strategies.

#### 3.2.3 The correlation between IL-10 and TGF-β1 expression and gastric cancer patient prognosis

**FIGURE 5:**
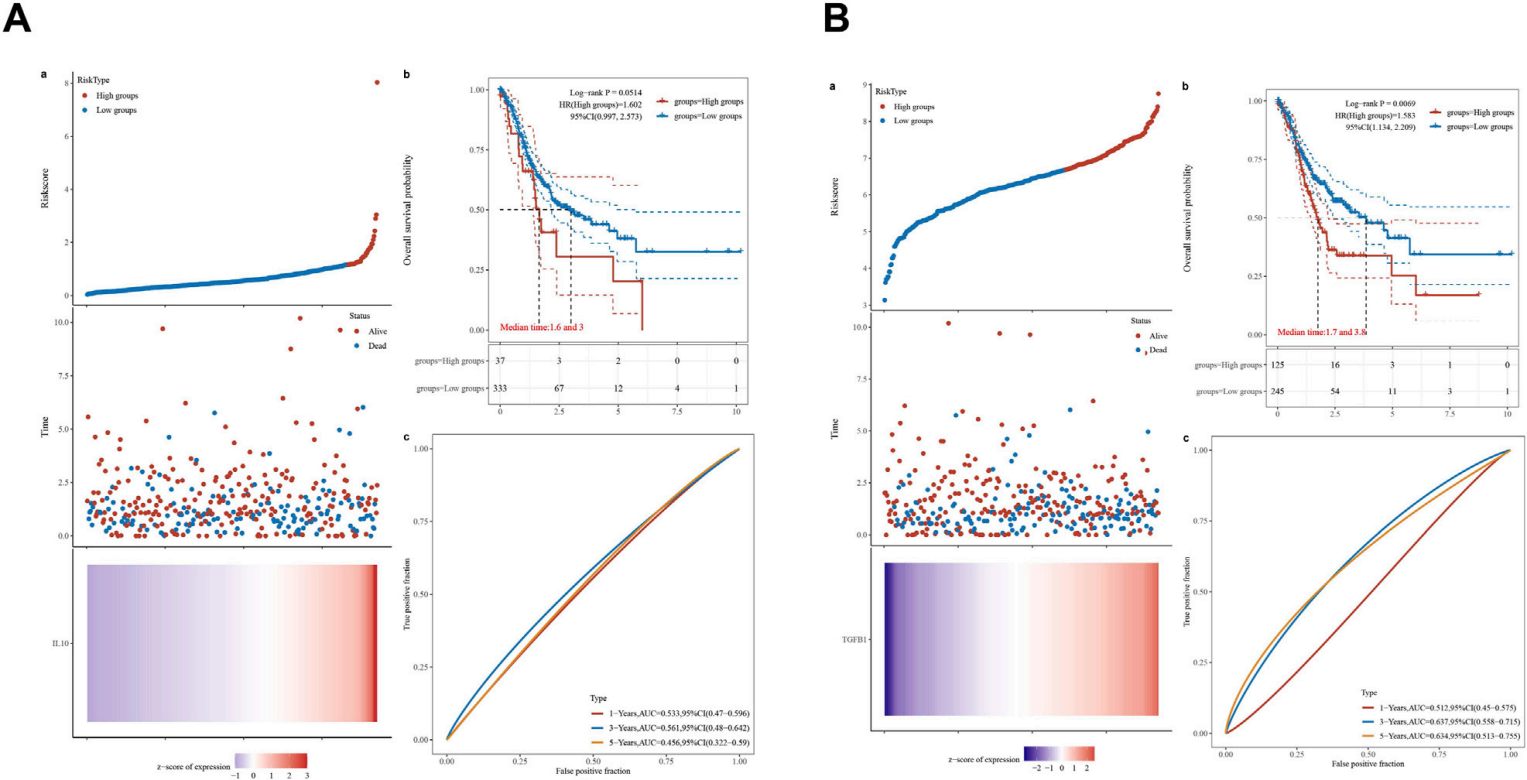
Different expression levels of IL-10** (A)** and TGFβ1** (B)** in patients with gastric cancer and their 5-year survival rate. **(a)** The relationship between gene expression and survival time and survival status in TCGA data. The top chart presents the distribution characteristics of the gene expression level from low to high in a scatter plot, with different colors marking different gene expression groups; the middle chart visually shows the relationship between the gene expression level of each sample and the corresponding patient’s survival time and survival status through the distribution of scatter plots; the bottom chart is a heat map of the gene expression in different samples. The x-axis of the top, middle, and bottom charts all represent samples, and the sample order is consistent. **(b)** The KM survival curve of the gene in TCGA data, where different groups are tested by log rank. HR (High exp) represents the risk coefficient of the high expression group relative to the low expression group samples. If HR > 1, it indicates that the gene is a risk factor (the higher the expression, the worse the prognosis); if HR < 1, it indicates that the gene is a protective factor (the higher the expression, the better the prognosis). 95% CI represents the confidence interval of HR. Median time represents the time corresponding to a 50% survival rate for both the high expression group and the low expression group (i.e., the median survival time), with units in years. **(c)** The ROC curve and AUC value of the gene at different times. The higher the AUC value, the stronger the predictive ability of the gene.

IL-10 expression and survival prognosis (Figure 5A): Using surv_cutpoint to categorize into high and low expression groups, the risk score plot indicates a more pronounced risk accumulation in the high expression group. The Kaplan-Meier analysis suggests a reduced median survival time for the high expression group, with a log-rank test yielding a P-value of 0.0514, nearing statistical significance. The ROC curve AUCs for 1, 3, and 5 years are 0.533, 0.561, and 0.456, respectively. Due to tumor heterogeneity and other factors, the predictive power of a single IL-10 indicator may be limited, and it is necessary to combine other immune factors for a comprehensive assessment.

TGF-β1 expression and survival prognosis (Figure 5B): Similarly, the high and low expression groups were divided. The risk score in the high expression group increased more significantly over time. In the Kaplan-Meier curve, the median survival time in the high expression group was significantly shortened (log-rank test *p* = 0.0069). The ROC curve AUC values are 0.512 for 1-year, 0.637 for 3-year, and 0.634 for 5-year. The predictive accuracy remains consistent over long-term periods of 3 and 5 years.

In summary, elevated TGF-β1 expression is strongly linked to reduced overall survival in gastric cancer patients, serving as a prognostic marker. Elevated IL-10 expression is linked to poor prognosis, though the association is marginal. Its value needs to be comprehensively judged in combination with multiple indicators of the immune microenvironment, providing a basis for prognosis stratification and multi-factor model construction of gastric cancer.

### 3.3 Single-cell transcriptome validation: expression characteristics of key molecules in gastric cancer immunity

**FIGURE 6:**
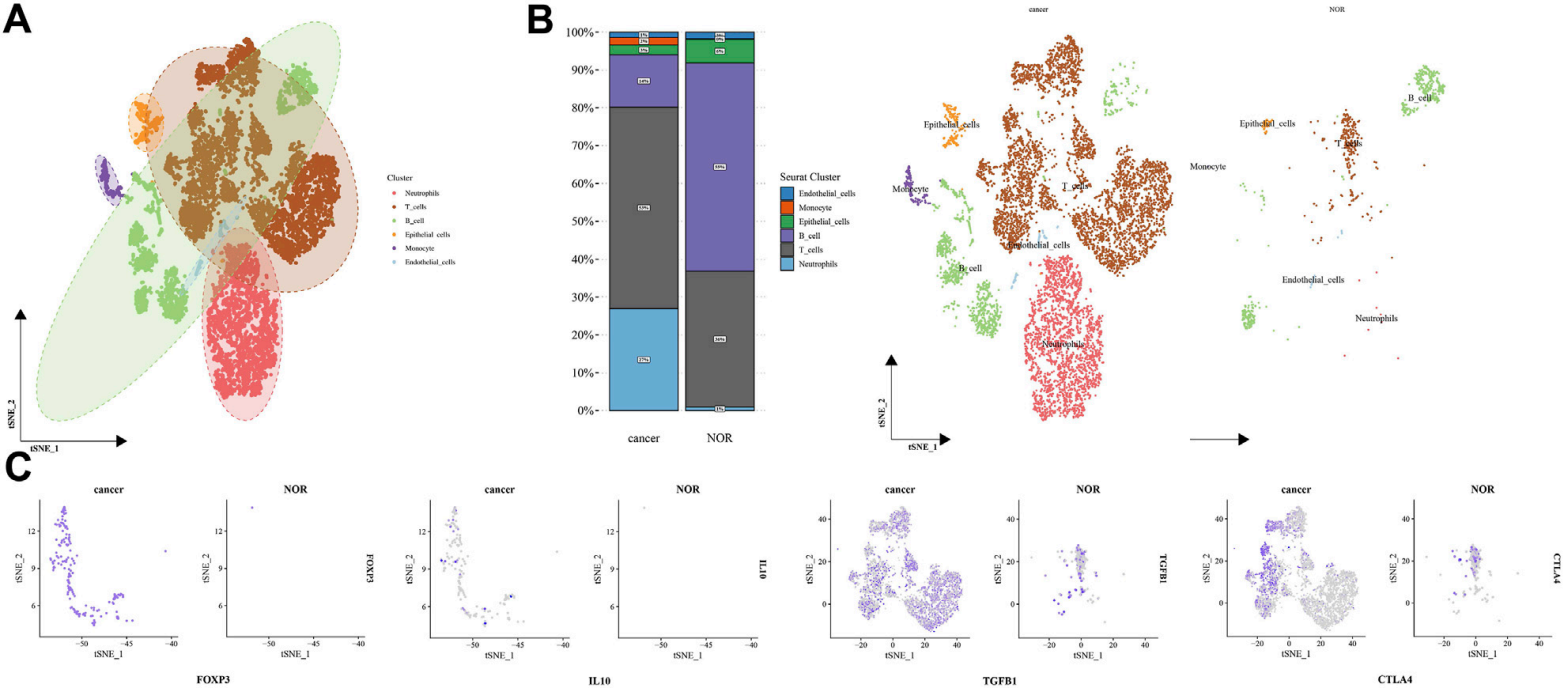
Single-cell analysis of gastric cancer dataset GSE163558. **(A)** The t-SNE plot of single-cell dimensionality reduction and clustering shows the distribution and clustering of various immune and epithelial cell subpopulations in gastric cancer (GC) and adjacent normal tissues (NOR), including T cells, B cells, macrophages, etc., with different colors and annotations. **(B)** The left side is a stacked bar chart showing the proportion of each cell subpopulation in gastric cancer (GC) and adjacent normal tissues (NOR) samples, and the right side is the visualization of the corresponding cell subpopulations in the t-SNE dimensionality reduction space, with typical cell types such as T cells, macrophages, and endothelial cells labeled. **(C)** The t-SNE plot of the expression distribution of key genes (FOXP3, IL1B, TGFB1, CTLA4, etc.) in single cells of gastric cancer (GC) and adjacent normal tissues (NOR), with purple dots representing cells with positive gene expression, demonstrating the expression differences and localization in different tissue microenvironments.

To bridge the gap between TCGA tissue data and animal models, we utilized the gastric cancer single-cell dataset GSE163558 (10× Genomics) from the GEO database. After quality control (filtering out cells with abnormal gene numbers and high mitochondrial content) using Seurat, normalization, and PCA analysis, we conducted cell clustering and annotation, focusing on the expression differences of Tregs and immune-related molecules (FOXP3, IL10, TGF-β1, CTLA4). The results are as follows:

Cell clustering and composition analysis revealed distinct subpopulations, including neutrophils, T cells, and B cells, using t-SNE dimensionality reduction (Figure 6A). In the tumor group, the proportions of T cells and neutrophils were significantly higher compared to the normal group, while epithelial cells were less prevalent (Figure 6B). This indicates altered immune cell infiltration in the gastric cancer microenvironment.

Differential expression of key immune molecules (Figure 6C). t-SNE visualization indicated a significantly higher distribution density of FOXP3, IL10, TGF-β1, and CTLA4 positive cells in the tumor group compared to the normal group, with gene expression concentrated in immune subpopulations like T cells. This indicates that in the gastric cancer microenvironment, Tregs-related molecules (FOXP3) and immune suppressive factors (IL10, TGF-β1), as well as checkpoint molecules (CTLA4), are highly expressed and enriched, jointly shaping an immunosuppressive microenvironment.

In conclusion, single-cell transcriptome validation shows that Tregs and immune suppressive-related molecules (IL10, TGF-β1, CTLA4) are highly expressed and enriched in immune cell subpopulations in gastric cancer tissues, consistent with the immunosuppressive phenotype of the tumor microenvironment. This compensates for the limitations of bulk RNA-seq data and provides a single-cell level basis for the exploration of immune regulatory mechanisms and therapeutic targets.

### 3.4 Body weight of mice

**FIGURE 7:**
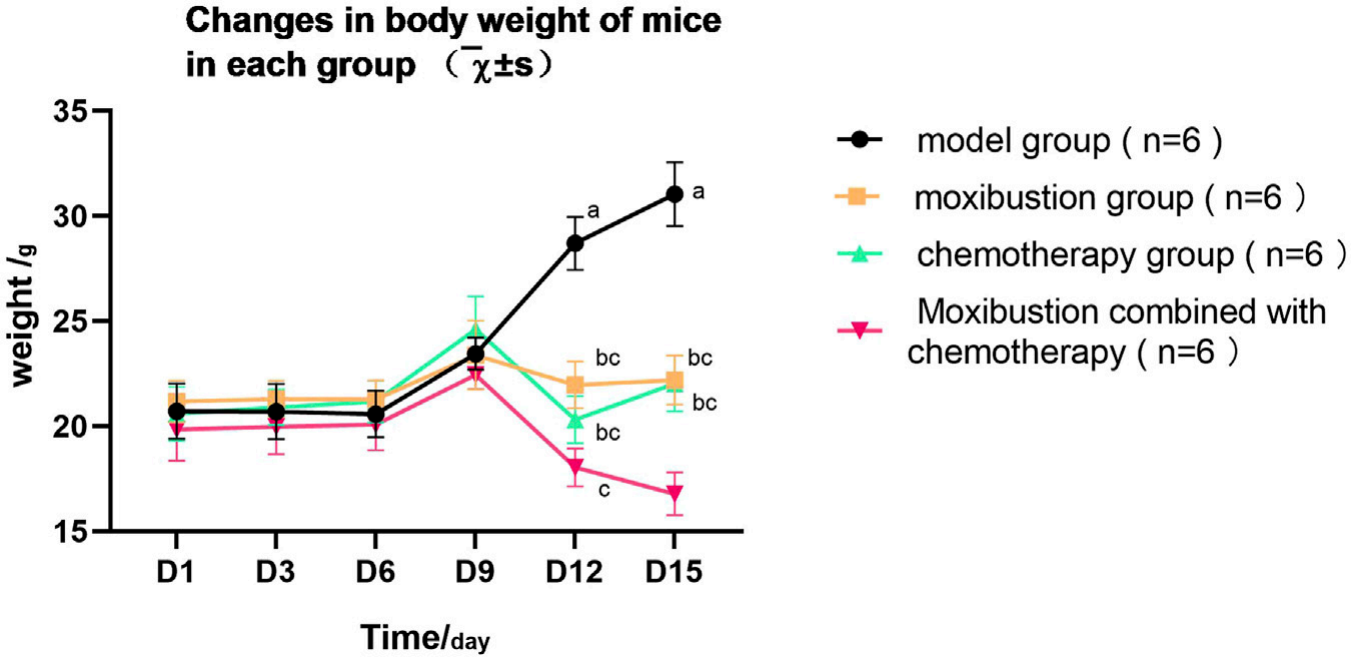
The changes in body weight of mice in each group. The horizontal axis represents the number of days of observation after the mouse model was established. The vertical axis indicates the body weight of the mice. The expression differences began to be shown from the 12th day as illustrated in the figure. The significant differences between groups are indicated by the letter method. If the letters are the same, it means there is no statistical difference between the two groups; otherwise, there is a difference (*p* < 0.05). **(a)** compared with the model group; **(b)** compared with the moxibustion group; **(c)** compared with the chemotherapy group. On the ninth, 12th, and 15th days, the body weights of each group were as follows: model group 23.45 ± 0.76 g, 28.7 ± 1.26 g, 31.04 ± 1.52 g. Moxibustion group 23.38 ± 1.64 g, 21.96 ± 1.11 g, 22.19 ± 1.16 g. Chemotherapy group 24.60 ± 1.57 g, 20.31 ± 1.12 g, 22.03 ± 1.32 g. Moxibustion combined with chemotherapy group 22.45 ± 0.69 g, 18.04 ± 0.90 g, 16.78 ± 1.03 g. There were significant differences in the effects of different interventions on the body weight changes of tumor-bearing mice. Repeated measures analysis of variance (ANOVA) revealed that the interaction effect between time and group was statistically significant (*p* < 0.001). The cumulative increase in body weight was 49.8%, 4.8%, and 7.0% in the model group, moxibustion group, and chemotherapy group, respectively. The body weight decrease in the moxibustion combined with chemotherapy group was 15.4%. Pairwise comparison of body weight on day 15 between groups showed that compared to the model group, the body weights of the moxibustion group (mean difference = −8.85 g, *p* < 0.001), chemotherapy group (mean difference = −9.01 g, *p* < 0.001), and combined group (mean difference = −14.26 g, *p* < 0 0.001) were significantly lower. The body weight of the combined group was also significantly lower than that of the moxibustion group (mean difference = −5.41 g, p < 0.001) and chemotherapy group (mean difference = −5.25 g, *p* < 0 0.001). Simple effect analysis of the time series indicated that the difference between the combined group and the model group was statistically significant from day 9 (*p* = 0 0.007) and maintained significance at subsequent time points (*p* < 0.001).

Repeated measures analysis of variance showed that the interaction effect between time and group was significant, *F* (15, 44.57) = 9.703, *p* < 0.001, partial η^2^ = 0.737. It showed that the trend of body weight change with time was different in different groups. As shown in Figure 7, during the first 9 days after modeling, the body weight of mice in all groups increased steadily without statistically significant differences (all *p* > 0.05). After that, the body weight of the model group continued to increase abnormally and was significantly higher than that of the other treatment groups on the 15th day.

In contrast, all the interventions showed a positive effect on body weight. Body weight in the moxibustion group remained relatively stable throughout the experimental period. The chemotherapy group and the moxibustion + chemotherapy combined group showed obvious weight inhibition effect, and the combined treatment group was the most prominent. At the end of the experiment (day 15), *post hoc* comparison showed that the body weight of the moxibustion + chemotherapy combined treatment group was significantly lower than that of the model group (the difference value was 14.258g, *p* < 0.001), the moxibustion group (the difference value was 5.413g, *p* < 0.001) and the chemotherapy group (the difference value was 5.253g, *p* < 0.001).

In conclusion, the difference in weight change was strongly related to the type of intervention. Moxibustion intervention can help to maintain weight stability, while chemotherapy and its combination therapy show stronger weight control effect, which provides an important behavioral basis for the subsequent analysis of tumor growth and inhibition rate.

### 3.5 Tumor mass and tumor inhibition rate in mice

**FIGURE 8:**
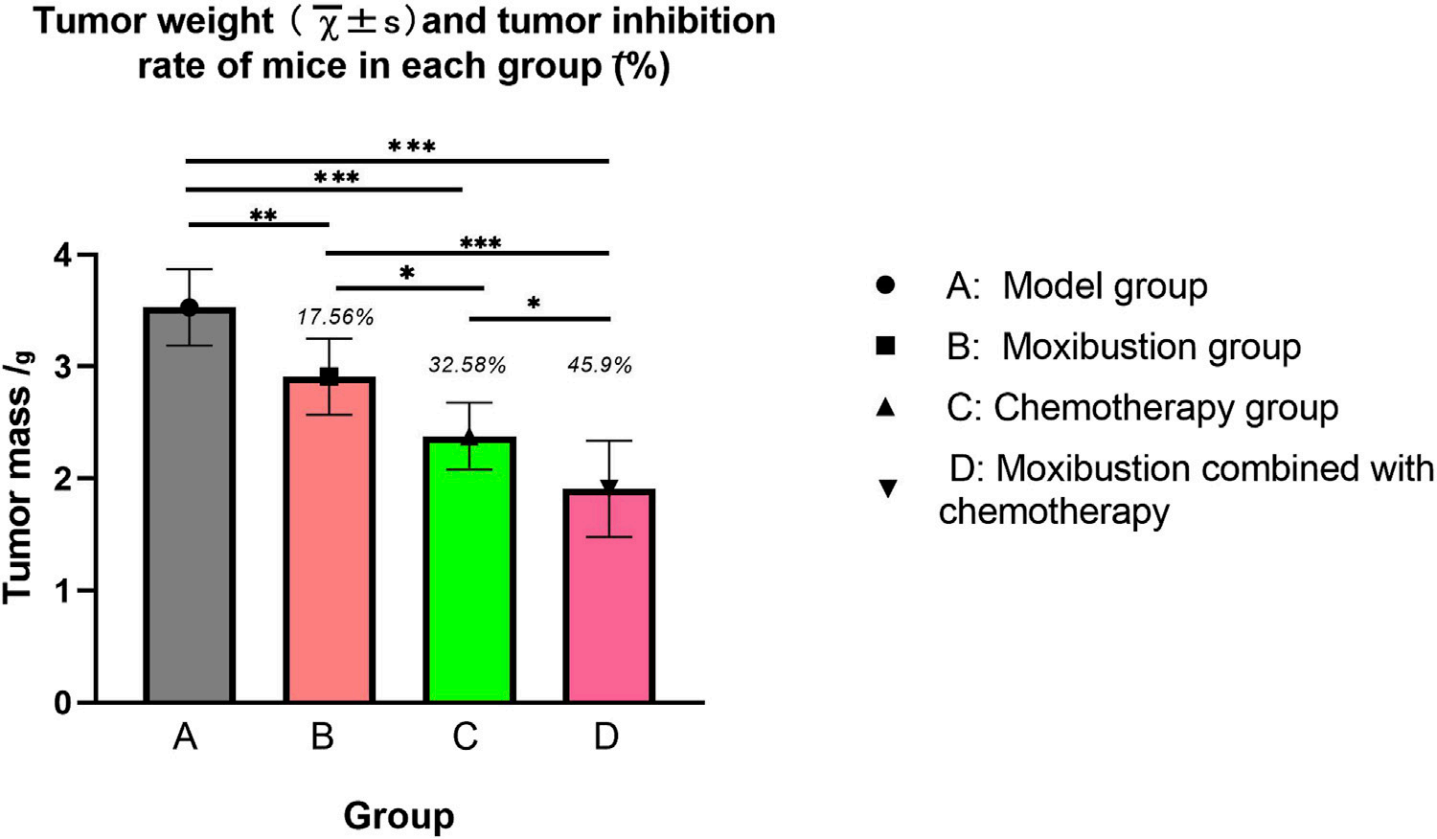
Tumor mass and tumor inhibition rate of each group of mice. The mean tumor weights of the four groups of mice were as follows: model group 3.53 ± 0.34 g, moxibustion group 2.91 ± 0.34 g, chemotherapy group 2.38 ± 0.30 g, and moxibustion combined with chemotherapy group 1.91 ± 0.43 g. The tumor inhibition rates were 17.56% for the moxibustion group, 32.58% for the chemotherapy group, and 45.90% for the moxibustion combined with chemotherapy group. The tumor weights of all groups decreased to varying degrees (* indicates *p* < 0.05, ** indicates *p* < 0.01, *** indicates *p* < 0.001).

To evaluate the effects of different intervention measures on tumor growth, we measured the tumor weights of mice in each group and calculated the tumor inhibition rate. The results showed (Figure 8) that compared with the model group, the tumor weights in the moxibustion group, chemotherapy group and the moxibustion combined with chemotherapy group were significantly reduced, with tumor inhibition rates of 17.56%, 32.58% and 45.9%, respectively. Notably, compared with the chemotherapy group alone, the tumor inhibition rate in the moxibustion combined with chemotherapy group was significantly higher (*p* = 0.002), indicating that the combination of moxibustion and chemotherapy produced a synergistic anti-tumor effect.

### 3.6 Multidimensional analysis of *in vivo* experiments in mice

**FIGURE 9:**
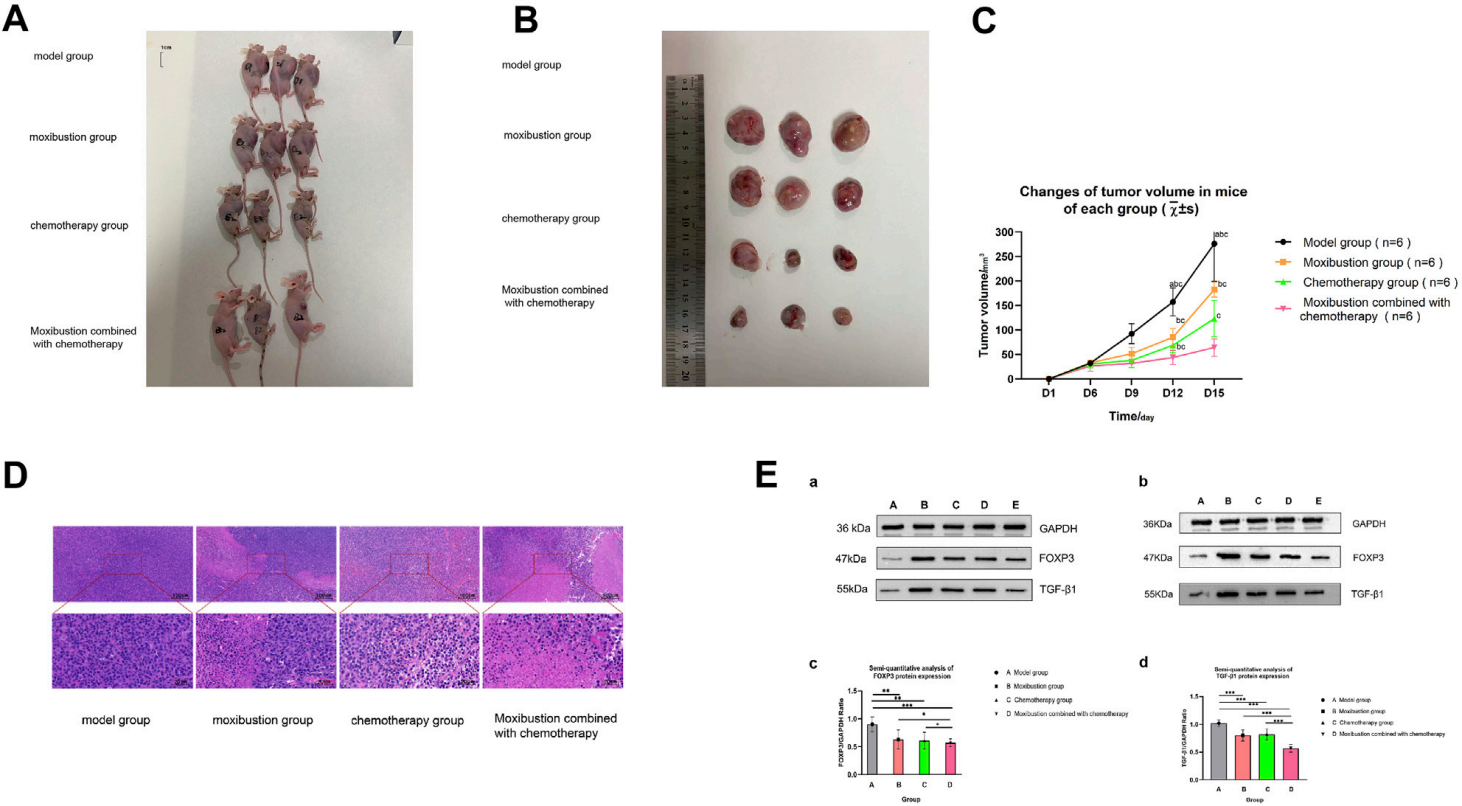
Experimental results in mice. **(A)** Grouped appearance images of nude mice, showing the general conditions of the model group, moxibustion group, chemotherapy group, and moxibustion combined with chemotherapy group. **(B)** Gross specimens of tumor tissues from each group of nude mice, comparing the size and shape differences of tumors in each group through a ruler. The two sub-figures have visual proportion differences due to different shooting distances; the actual size should be based on the scale and quantitative data. **(C)** The changes in tumor volume of each group of mice. The horizontal axis represents the number of days of observation after mouse modeling, and the vertical axis indicates the size of the tumor volume. The expression differences began to be shown from the 12th day as illustrated in the figure. The significant differences between groups are indicated by the letter method. If the letters are the same, it means there is no statistical difference between the two groups; otherwise, there is a difference (p < 0.05). **(A)** compared with the model group; **(B)** compared with the moxibustion group; **(C)** compared with the chemotherapy group. Model group: 32.01 ± 5.49 mm^3^, 92.19 ± 20.40 mm^3^, 157.30 ± 28.79 mm^3^, 276.10 ± 76.86 mm^3^. Moxibustion group: 32.96 ± 2.93 mm^3^, 51.29 ± 12.55 mm^3^, 85.08 ± 17.83 mm^3^, 182.55 ± 15.40 mm^3^. Chemotherapy group: 30.36 ± 7.72 mm^3^, 37.97 ± 15.11 mm^3^, 68.57 ± 15.09 mm^3^, 123.15 ± 36.76 mm^3^. Moxibustion combined with chemotherapy group: 26.39 ± 10.52 mm^3^, 31.50 ± 8.50 mm^3^, 43.53 ± 14.24 mm^3^, 64.09 ± 17.86 mm^3^. There were significant differences in tumor volume among the groups of mice (overall between-group effect: F (3,20) = 90.502, p < 0.001, partial η^2^ = 0.931). Post hoc comparisons, with the moxibustion plus chemotherapy group as the reference, showed that on the ninth day, the tumor volume of the model group and the moxibustion group was significantly larger than that of the moxibustion plus chemotherapy group (both p < 0.05); by the 12th day, the tumor volume of the chemotherapy group was also significantly larger than that of the moxibustion plus chemotherapy group (p = 0.041); on the 15th day, the tumor volume of all single-treatment groups was extremely significantly larger than that of the moxibustion plus chemotherapy group (model group vs. combined group: p < 0.001; moxibustion group vs. combined group: p < 0.001; chemotherapy group vs. combined group: p = 0.031). **(D)** HE staining pathological sections of tumor tissues from each group (upper: low-power field; lower: high-power field of the selected area). **(E)** Protein expression of FOXP3 and TGF-β1 in tumor tissues. (a) Protein molecular weight standard. (b) Representative results of three independent repeated experiments detected by Western blot. The letters A-E above the bands represent the blank control group, model group, moxibustion group, chemotherapy group, and combined treatment group, respectively. (c–d) Semi-quantitative statistical analysis of FOXP3 (c) and TGF-β1 (d) protein expression. The data were calculated by the ratio of the gray value of the target protein to GAPDH and expressed as mean ± standard deviation. *p < 0.05, **p < 0.01, ***p < 0.001 (α = 0.05).

Macroscopic examination of the tumor (Figures 9A,B) revealed that the model group of nude mice exhibited large, irregularly shaped tumors. The moxibustion group showed reduced tumor volume compared to the model group, while the chemotherapy group had even smaller tumors. The smallest tumor volume was observed in the group receiving combined moxibustion and chemotherapy, indicating the enhanced efficacy of the combined treatment in inhibiting tumor growth.

The volume of the tumor in mice (Figure 9C).

To analyze the effects of different intervention measures on tumor growth, we monitored the tumor volume of four groups of mice for 15 days. Repeated measures ANOVA showed a significant interaction effect between time and group, F (15, 44.570) = 9.703, p < 0.001, partial η^2^ = 0.737, indicating that the tumor growth trends of each group were different. The overall between-group effect was also highly significant, F (3, 20) = 90.502, p < 0.001, partial η^2^ = 0.931.

At the early stage of the experiment (day 6), there was no statistically significant difference in tumor volume among the groups (all p > 0.05). On the ninth day, the tumor volumes in the model group and the moxibustion group were significantly larger than those in the combined treatment group (all p < 0.05); by the 12th day, the tumor volume in the chemotherapy group was also significantly larger than that in the combined treatment group (p = 0.041); by the end of the experiment (the 15th day), the tumor volumes in all single treatment groups were extremely significantly larger than those in the combined treatment group (model group vs. combined group: p < 0.001; moxibustion group vs. combined group: p < 0.001; chemotherapy group vs. combined group: p = 0.031). Specifically, the tumor in the model group continued to grow rapidly, reaching a volume of (276.10 ± 76.86) mm^3^ by the 15th day. All treatment groups showed tumor-suppressing effects, with the strength of the effects in the following order: moxibustion + chemotherapy group > chemotherapy group > moxibustion group. By the 15th day, the tumor volumes in the moxibustion group, the chemotherapy group, and the combined treatment group were (182.55 ± 15.40) mm^3^, (123.15 ± 36.76) mm^3^, and (64.09 ± 17.86) mm^3^, respectively. The results indicated that the combination of moxibustion and chemotherapy produced a synergistic enhancement effect in inhibiting tumor growth, which was significantly superior to any single therapy.

Pathological morphology of tumor tissue (Figure 9D): HE staining showed that tumor cells were present in all groups. In the model group, the tumor cell density was high and the arrangement was tight, with no obvious necrotic foci; in the moxibustion group, chemotherapy group, and moxibustion combined with chemotherapy group, varying degrees of tumor cell death were observed, with enlarged cell nuclei and reduced cell division. Among them, the tumor cell density in the moxibustion combined with chemotherapy group decreased significantly, and a large number of dead cells appeared, indicating that the combined intervention had a more prominent effect on improving the pathological morphology of tumor tissue.

To objectively quantify the above morphological changes, we further conducted a semi-quantitative analysis of tumor necrosis and cell density.

The semi-quantitative pathological scoring results of tumor tissues showed that there were significant statistical differences among the groups in terms of the range of necrotic areas (chi-square value = 16.862, *p* = 0.001) and tumor cell density (chi-square value = 18.354, *p* < 0.001). Given that six pairwise comparisons were conducted, the Bonferroni method was used to correct the significance level, setting the corrected α' = 0.0083. Post hoc pairwise comparison analysis indicated that for the necrotic area, only the difference between the model group and the combined group remained statistically significant after correction (Z = −3.207, *p* = 0.001). For tumor cell density, the comparisons between the model group and the chemotherapy group, as well as the model group and the combined group, remained statistically significant after correction (*p* = 0.004 for both). There was a trend of benefit for the chemotherapy group compared to the combined group (z = −1.925, *p* = 0.054).

Under the strict control of type I error, the results demonstrated that compared with the model group, the combination of moxibustion and chemotherapy could significantly induce a wider range of tumor necrosis and significantly reduce the tumor cell burden, confirming the anti-tumor effect of this combined regimen at the pathological level.

Expression of immune-related proteins (Figure 9E): The expression levels of immune suppressive-related proteins FOXP3 and TGF-β1 in tumor tissues were detected by Western blot (9E a-b). Semi-quantitative analysis indicated that the relative expression levels of FOXP3 and TGF-β1 proteins (9E c-d) in the moxibustion group, chemotherapy group and combined treatment group were significantly lower than those in the model group. The expression of FOXP3 protein: the model group (0.90 ± 0.13) was significantly higher than the moxibustion group (0.63 ± 0.17, p = 0.003), the chemotherapy group (0.61 ± 0.15, *p* = 0.002) and the combined treatment group (0.57 ± 0.07, *p* < 0.001). Additionally, the expression level in the combined treatment group was significantly lower than that in the moxibustion group (*p* = 0.023) and the chemotherapy group (*p* = 0.039), while there was no statistical difference between the moxibustion group and the chemotherapy group (*p* = 0.807). The expression of TGF-β1 protein: the model group (1.02 ± 0.062) was significantly higher than the moxibustion group (0.80 ± 0.10, *p* < 0.001), the chemotherapy group (0.82 ± 0.10, *p* < 0.001) and the combined treatment group (0.57 ± 0.07, *p* < 0.001). The expression level in the combined treatment group was also significantly lower than that in the moxibustion group and the chemotherapy group (both *p* < 0.001), while there was no statistical difference between the moxibustion group and the chemotherapy group (*p* = 0.664). The results showed that both moxibustion and chemotherapy could effectively downregulate the key proteins in the tumor immune suppressive microenvironment, and the combined treatment produced a synergistic enhancement effect.

In conclusion, moxibustion combined with chemotherapy was more effective than single moxibustion or chemotherapy in inhibiting tumor growth in mice, improving the pathological morphology of tumor tissue, and down-regulating the expression of immune suppressive proteins.

**FIGURE 10:**
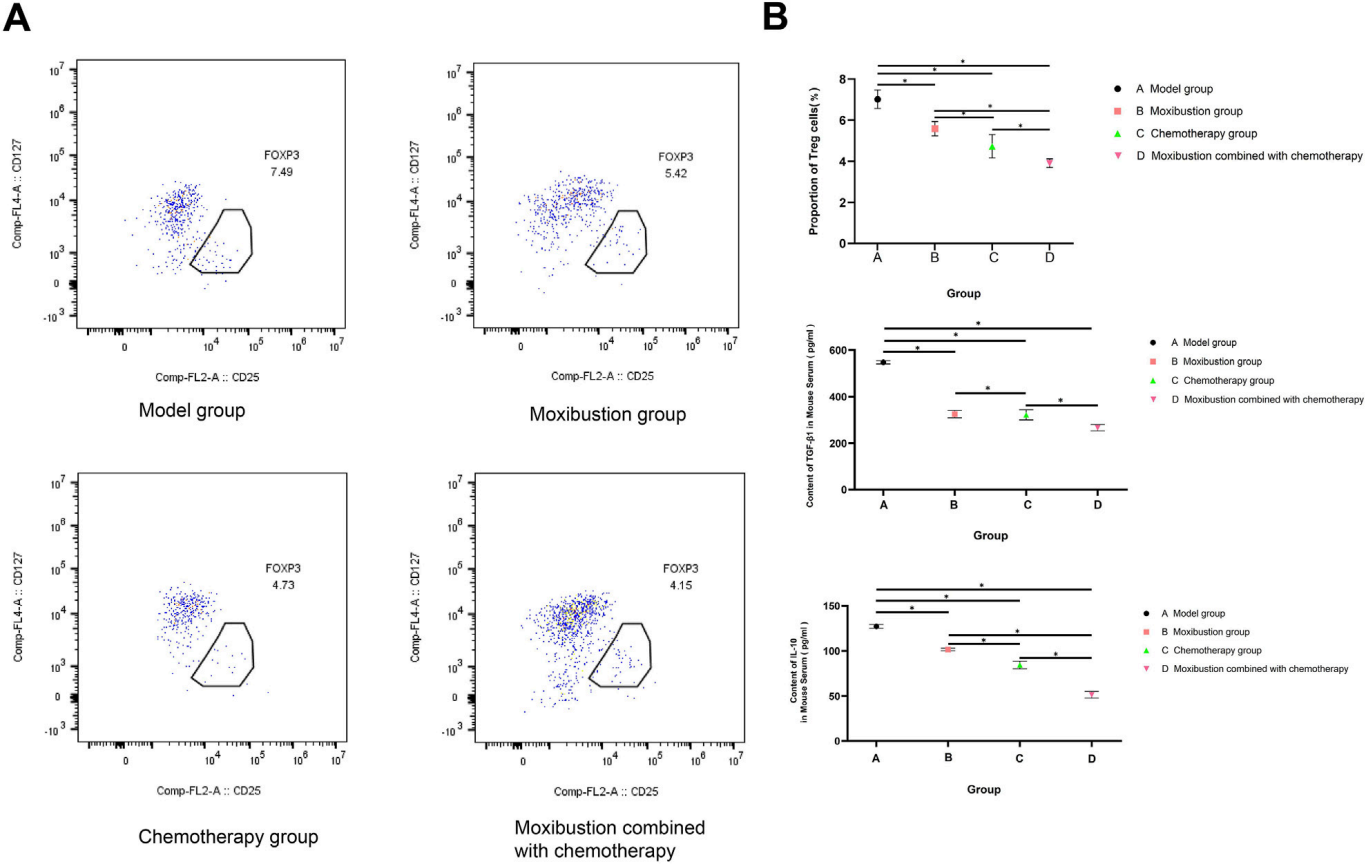
The effects on the expression of FOXP3+ Treg in the tumor microenvironment and key inhibitory factors in the peripheral blood of mice in each group. **(A)** Representative flow cytometry dot plots of the proportion of CD4^+^CD25+FOXP3+ regulatory T (Treg) cells in the peripheral blood of each group of mice. The cell gating strategy was based on the consensus marker scheme for Treg cell analysis: first, the lymphocyte population was gated, followed by gating of CD4^+^ T cells and CD25^+^ cells in sequence, and finally the Treg cell population was determined by FOXP3+ cells. The model group was 7.49%, the moxibustion group was 5.42%, the chemotherapy group was 4.73%, and the moxibustion combined with chemotherapy group was 4.15%. **(B)** Comparison of CD4 + CD25 + FOXP3 + Treg, TGF-β1 and IL-10 levels in the serum of mice among different groups. A, B, C, and D represent different groups. The expression levels of Treg (%) in the model group, moxibustion group, chemotherapy group, and moxibustion combined with chemotherapy group were 7.02 ± 0.45, 5.59 ± 0.35, 4.73 ± 0.57, and 3.91 ± 0.21, respectively; the expression levels of TGF-β1 (pg/mL) were 547.84 ± 7.25, 325.24 ± 15.03, 322.05 ± 21.91, and 266.82 ± 13.71, respectively; the expression levels of IL-10 (pg/mL) were 127.21 ± 2.07, 101.68 ± 1.42, 84.53 ± 4.25, and 51.42 ± 3.65, respectively.

The expression of FOXP3^+^ Treg in the tumor microenvironment and the main inhibitory factors in the peripheral blood across different mouse groups: The results (Figure 10) show that the proportion of Treg cells (7.02% ± 0.45%), TGF-β1 (547.84 ± 7.25 pg/mL), and IL-10 (127.21 ± 2.07 pg/mL) in the model group were at the highest levels compared to the other groups. Compared with the model group, all treatment groups could significantly reverse this immunosuppressive state: the proportions of Treg cells, TGF-β1, and IL-10 in the moxibustion group, chemotherapy group, and combined treatment group were all significantly reduced (*p* < 0.01 for IL-10 among all groups). Notably, the combined treatment group had the most thorough inhibitory effect on all indicators, with Treg cell proportion (3.91% ± 1.21%), TGF-β1 (266.82 ± 13.71 pg/mL), and IL-10 (51.42 ± 3.65 pg/mL) levels significantly lower than those in the model group (*p* < 0.001) and also significantly lower than those in the moxibustion group and chemotherapy group (all *p* < 0.01). Additionally, although the moxibustion group and chemotherapy group had similar effects in reducing TGF-β1 levels (*p* = 0.723), the combined treatment group showed a significant synergistic enhancement effect compared to either single treatment group.

**FIGURE 11:**
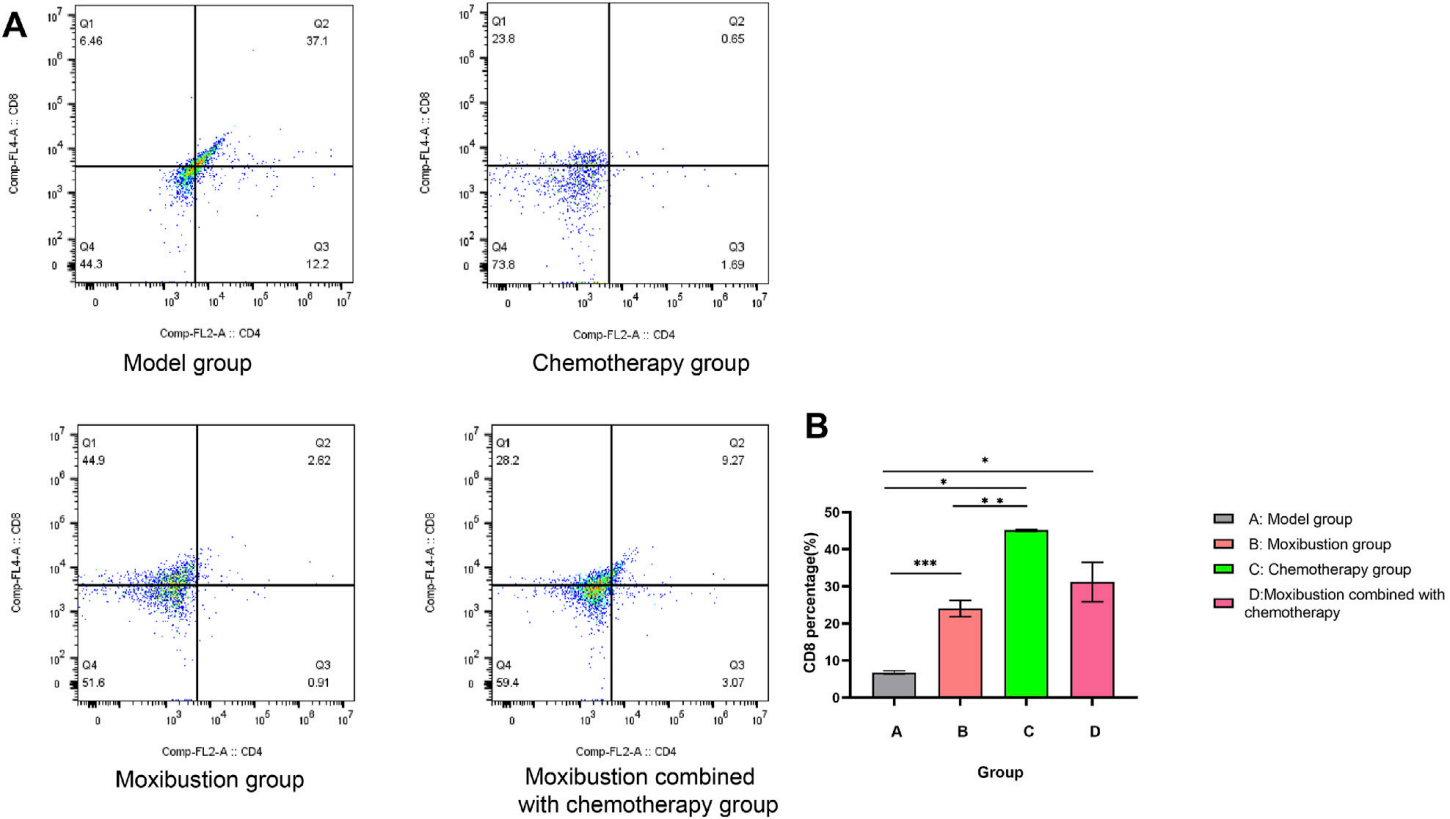
Quadrant Analysis of CD8^+^ T Cells via Flow Cytometry. **(A)** CD4/CD8 two-parameter flow cytometry quadrant analysis of CD8^+^T cell subsets. Among them, Comp-FL2-A, a CD4 fluorescent signal channel, was used to define CD8^+^T cell population. Q1 to Q4 represent four quadrants corresponding to cell subsets with different CD4/CD8 expression profiles, and the percentage of cells in each quadrant has been annotated in figure. **(B)** The results of pairwise comparison showed that: Compared with the model group, the proportion of CD8^+^T cells in the chemotherapy group (*p* = 0.010), the moxibustion combined with chemotherapy group (*p* = 0.037) and the moxibustion group (*p* < 0.001) were significantly different. Compared with the chemotherapy group, the proportion of CD8^+^T cells in the moxibustion group was significantly different (*p* = 0.008), while there was no significant difference between the chemotherapy group and the moxibustion combined with chemotherapy group (*p* = 0.329). There was no significant difference in the proportion of CD8^+^T cells between the moxibustion combined with chemotherapy group and the moxibustion group (*p* = 0.111). *P < 0.05, **P < 0.01, ***P < 0.001 (α = 0.05).

The infiltration level of CD8^+^ T cells in the tumor microenvironment: The quantitative analysis results showed that (Figure 11) compared with the model group (6.46%), all treatment groups could significantly increase the level of CD8^+^ T cells: the moxibustion group (28.2%, *p* < 0.001), the chemotherapy group (23.8%, *p* = 0.010), and the moxibustion combined with chemotherapy group (22.8%, *p* = 0.037). Notably, the moxibustion group had the most significant promoting effect, with a significantly higher proportion of CD8^+^ T cells than the chemotherapy group (*p* = 0.008). However, there were no significant differences between the combined treatment group and the chemotherapy group (*p* = 0.329) or the moxibustion group (*p* = 0.111). The results indicate that both moxibustion and chemotherapy can effectively promote the infiltration of CD8^+^ cytotoxic T cells in tumor tissues. Among them, moxibustion alone shows the strongest promoting effect, while the combined intervention does not show a synergistic enhancing effect under the experimental conditions of this study.

The results demonstrated that moxibustion combined with chemotherapy has a bidirectional immunomodulatory effect on the tumor immune microenvironment. This combined regimen not only effectively downregulates immunosuppressive components (including Treg cells and key cytokines TGF-β1 and IL-10), but also significantly enhances the levels of cytotoxic CD8^+^ T cells. Notably, while moxibustion alone showed the most potent effect in expanding CD8^+^ T cells, the combined treatment did not yield a further synergistic increase in this specific subset. This underscores the complexity of immune modulation. The superior anti-tumor efficacy of the combination likely stems from its broader and more balanced immunomodulatory profile, achieving the most potent suppression of immunosuppressive components alongside a significant enhancement of cytotoxic T cell infiltration compared to the model, thereby effecting a more favorable overall remodeling of the tumor immune landscape.

## 4 Discussion

Gastric cancer can be classified under the categories of “Fu Liang”, “Fan Wei”, “Ji Ju”, and “Zheng Xia” in traditional Chinese medicine (MEDICINE, C. A. O. C., 2008). As early as in “Ji Sheng Fang”, there were detailed records: “The appearance of Fu Liang starts from below the navel. dry throat, heart irritation, and in severe cases, hematemesis, causing poor appetite and emaciation” (Yan and Ji Sheng, 2012). These symptoms are similar to those of advanced gastric cancer, such as hematemesis, anorexia, and emaciation. Traditional medical theory attributes gastric cancer primarily to a decline in the body’s vital energy, which permits the invasion of pathogenic factors. Gu Ben Pei Yuan Moxibustion integrates the Gu Ben Pei Yuan concept with moxibustion in a novel approach. By applying moxibustion to acupoints with the function of Gu Ben Pei Yuan, the therapeutic goal is achieved. In “Research Integration of Xin’an Medicine”, it is stated that “Gu Ben Pei Yuan means to strengthen the innate foundation and nourish the acquired essence” (Jian, 2018). The study selected the following acupoints: Zhong Wan, to regulate qi, relieve stagnation, and harmonize the spleen and stomach; Zusanli, to tonify qi, strengthen the spleen, dry dampness, transform phlegm, and enhance immunity; Guanyuan, to warm yang, assist fire, and nourish the innate foundation; and Qihai, to tonify qi, lift yang, and strengthen the kidney and spleen. The combined moxibustion of these four acupoints aims to nourish qi and blood and replenish the vital energy, embodying the concept of Gu Ben Pei Yuan.

In recent years, extensive research has been conducted both domestically and internationally on the physical and medicinal effects of moxibustion, its impact on the circulatory system and immune system, and its regulation of the body’s metabolism (Lu et al., 2023; Hu et al., 2021; Wang et al., 2025). Currently, there are no clinical studies reporting that moxibustion alone can directly inhibit tumor growth. However, animal studies have confirmed that grain-sized moxibustion at Zusanli can improve the survival status of Lewis lung cancer mice, reduce the mortality rate, and control the growth rate of the tumor (Hu et al., 2021). Zhang Feicheng et al. A study on breast cancer-bearing mice revealed that moxibustion combined with chemotherapy significantly reduced tumor volume compared to the model group, with the combination treatment showing the most pronounced effect, suggesting a synergistic interaction in inhibiting tumor growth (Fei-Cheng et al., 2025). The experiment revealed no initial significant differences in the body weight of mice across all groups. From the ninth day onward, the body weight of mice in each treatment group decreased, whereas the model group exhibited continued weight gain. This phenomenon might be linked to the nature of tumors as consumptive diseases. During treatment, the drugs’ inhibitory impact on tumor growth likely contributed to a reduction in the mice’s body weight. The model group exhibited significantly higher tumor volume and mass compared to other groups, reinforcing the inhibitory effects of moxibustion and chemotherapy on tumor growth. This study found that moxibustion combined with chemotherapy not only inhibits Treg cells and immunosuppressive cytokines but also shows a trend of increasing the proportion of CD8^+^ CTLs. This suggests that its mechanism of action may be a “bidirectional immunomodulation,” simultaneously attenuating immunosuppression and enhancing immune attack. The “guben peiyuan ” moxibustion method may improve the “deficiency and exhaustion” state caused by tumors by regulating the neuro-endocrine-immune network, creating a more favorable immune environment for chemotherapy. Future research can explore its effects on antigen-presenting cells (such as dendritic cells), myeloid-derived suppressor cells (MDSCs), or other checkpoint molecules (such as PD-1/PD-L1) to more comprehensively clarify its systemic regulatory role.

Tumors can prompt CD4^+^ T cells to transform into CD4^+^CD25+FOXP3+ Treg cells, crucial in the progression of autoimmune diseases and tumors. Research indicates that Treg cells suppress the immune system, diminishing the body’s ability to surveil and attack tumor cells, which facilitates tumor immune escape (Hosseinalizadeh et al., 2023). Research suggests that combining cryoablation with a traditional Chinese lung-nourishing formula may decrease CD4^+^ CD25^+^ Foxp3+ Treg cell proportions in the spleen and reduce Foxp3 expression in tumor tissues, thereby inhibiting Lewis lung cancer proliferation (Xiao et al., 2023). Our experiment demonstrated that moxibustion and chemotherapy both decreased the percentage of CD4^+^CD25+Foxp3+ Treg cells in mice’s peripheral blood. While chemotherapy alone had a greater impact, combining it with moxibustion more effectively reduced the proportion of Treg cells. The findings indicate that integrating moxibustion with chemotherapy could improve the anti-tumor immune response by modulating Treg cells.

TGF-β is a multifunctional cytokine involved in regulating cellular processes such as proliferation, differentiation, apoptosis, inflammation, and tumor progression (Luo et al., 2025). In various cancers, TGF-β1 is frequently overexpressed, leading to disrupted signal transduction. Initially, it can suppress tumor growth; however, in later stages, it tends to promote tumor progression, thereby enhancing the malignant phenotype (Garg et al., 2025; Liu et al., 2021). TGF-β1 levels are significantly lower in healthy tissues compared to gastric cancer tissues. As gastric cancer progresses, characterized by deeper cell invasion, more extensive lymphatic metastasis, or advanced stages, TGF-β1 content increases (Liu et al., 2025). IL-10 is an immunosuppressive cytokine involved in immune regulation, angiogenesis, and significantly influences tumor growth and metastasis by directly affecting both tumor and immune cells (Jung et al., 2023). Huang Wenjuan (Huang et al., 2014)et al. found that moxibustion intervention in tumor-bearing mice could help correct the Th1/Th2 imbalance caused by tumors by reducing IL-10 levels, thereby possibly enhancing the body’s anti-tumor immune function. The experiment demonstrated that moxibustion combined with chemotherapy significantly decreased serum IL-10 and TGF-β1 levels in gastric tumor-bearing mice compared to the model group. Furthermore, this combination was more effective in reducing these levels than chemotherapy alone. Moxibustion may boost immune function and significantly contribute to tumor adjuvant therapy by inhibiting tumor invasion and metastasis.

This study has multiple advantages: Firstly, it established a three-level research system of “pan-cancer bioinformatics analysis - gastric cancer clinical data mining - animal experiment verification”, achieving a deep connection from the population to the individual and from the clinical to the basic level. Pan-cancer analysis identified the abnormal upregulation of immune suppressive factors IL-10 and TGF-β1 across various cancers, highlighting their potential as universal therapeutic targets. TCGA gastric cancer data confirmed a positive correlation between Foxp3 and IL-10, TGF-β1 expression, and linked these molecules to poor prognosis in gastric cancer patients. Additionally, the single-cell dataset GSE163558 demonstrated the enrichment of tumor-local Tregs and immune suppressive molecules, underscoring the significance of the immune suppressive microenvironment in gastric cancer development from multiple perspectives.

Secondly, the mechanism demonstration formed a relatively complete closed loop. This study, through multi-platform experimental data, jointly reveals that moxibustion combined with chemotherapy can significantly improve the tumor immune microenvironment. Specifically, at the systemic level, flow cytometry and ELISA detection confirm that the combined treatment significantly reduces the proportion of Treg cells in peripheral blood and the levels of serum immunosuppressive factors IL-10 and TGF-β1; at the tissue molecular level, Western blot analysis further shows that the protein expression of TGF-β1 and its downstream key functional molecule Foxp3 in tumor local tissues is also simultaneously downregulated. Most importantly, these results logically form a coherent mechanism chain: the combined treatment may directly or indirectly inhibit TGF-β1 in the tumor microenvironment, thereby weakening the expression of Foxp3, the “master switch” that drives the differentiation of naive T cells into Treg cells, ultimately leading to a reduction in Treg cells at both systemic (peripheral blood) and local (tumor tissue) levels and the alleviation of the overall immunosuppressive state. Therefore, the coordinated downregulation of TGF-β1 and its downstream functional marker Foxp3, observed both systemically and within the tumor tissue, strongly suggests that targeting this axis may constitute a core mechanism underlying the Treg-modulating and immune-remodeling effects of the combined therapy.

Thirdly, it achieved innovative breakthroughs from the perspective of integrating traditional Chinese and Western medicine. For instance, although the proportion of CD8^+^ in the combined group was slightly lower than that in the moxibustion group, it achieved the best balance in the most crucial comprehensive indicators: the highest tumor inhibition rate (45.9%), the lowest decrease in Treg proportion (4.15%), and the most significant improvement in pathology. This indicates that the advantage of the combined therapy does not lie in the extremization of a single indicator, but in the multi-target and multi-pathway immune system reprogramming (Immune Reprogramming), thereby achieving the best overall anti-tumor effect. The findings of this study have potential clinical translational value. On the basis of the existing standardized chemotherapy for gastric cancer, the adjunctive use of moxibustion guided by the principle of ‘consolidating the root and nourishing the essence’ may become a feasible strategy for integrating traditional Chinese and Western medicine. In clinical practice, moxibustion can be considered to be applied at specific acupoints (such as Zusanli and Zhongwan) during the chemotherapy intervals, aiming to alleviate the toxic and side effects of chemotherapy (such as myelosuppression and fatigue), while potentially enhancing the anti-tumor efficacy by regulating the immune microenvironment.

However, the study also has certain limitations. Firstly, the lack of a sham moxibustion control group prevents us from definitively distinguishing the specific effects of moxibustion at the selected acupoints from non-specific thermal effects. Secondly, the use of T-cell deficient nude mouse models, while essential for ensuring reliable tumor engraftment, represents a fundamental constraint on this study (Inoue et al., 2023; Hiraga et al., 2024; Gibbs et al., 2022). This model limits our ability to observe a complete adaptive immune response. Although we implemented a multi-level analytical strategy—by examining tumor tissue proteins and systemic cytokine levels—to compensate for this limitation, the immunological parameters measured (such as Treg and CD8^+^ T cell proportions, which likely originate from residual or underdeveloped lineages) must be interpreted with caution regarding their absolute values. Consequently, our findings should be positioned as a robust proof-of-concept demonstrating the therapeutic potential and immunomodulatory trend of the combined regimen (Li et al., 2023; Kostecki et al., 2025). We anticipate that in hosts with intact immune function, the efficacy of the combined therapy in activating cytotoxic lymphocytes and remodeling the immunosuppressive microenvironment could be even more pronounced than what was observed here (Sen et al., 2025; Zahid Hosen et al., 2025), a hypothesis that underscores the need for future validation in immunocompetent models. Third, the discrepancy between clinical data (mRNA levels) and animal experimental data (protein levels) indicates that what we have observed is a correlation rather than an exact equivalence of biological processes. Although this ‘level difference’ does not affect the validity of the core conclusion of this study, it suggests that there is complexity in the post-transcriptional regulation, protein secretion, and systemic effects of IL-10/TGF-β1 in the future, multi-level detection can be conducted simultaneously in clinical samples to enhance data consistency.

Moreover, current research primarily involves correlation analysis and lacks blocking experiments on IL-10 and TGF-β1 to directly establish their causal relationship with the tumor immune microenvironment and treatment outcomes. Future research could employ gene knockout, antibody blocking, and similar techniques to investigate the mechanisms of key molecules. Finally, the sample size in each group of the mouse experiments was relatively small which may have an impact on statistical power. Future studies can increase the sample size to obtain more convincing experimental results. The observation period of this study was designed to focus on the early regulatory effects of treatment on the tumor immune microenvironment. Future research should be dedicated to validating these findings in immunocompetent animal models, incorporate sham moxibustion control groups (Sun et al., 2024; Wang et al., 2022), analyze individual biological replicates, optimizing the dosage (such as different stimulation durations), and the treatment course (such as short, medium, and long-term interventions), and prospectively collecting serial serum samples for comprehensive cytokine analysis (such as IFN-γ, TNF-α, and IL-12, etc.) as well as long-term survival studies. Ultimately, this will drive the design of rigorous clinical trials to obtain more convincing experimental results to evaluate the safety and efficacy of this integrated traditional Chinese and Western medicine strategy in gastric cancer patients, which is an important next step in verifying its clinical translational value. Despite these shortcomings, this study has revealed the role of immune suppressive microenvironment-related molecules in gastric cancer and the intervention effect of moxibustion combined with chemotherapy from multiple dimensions, laying a foundation for subsequent research.

## 5 Conclusion

Moxibustion combined with chemotherapy effectively suppresses gastric cancer growth in mice. This combined therapy downregulates Foxp3, IL-10, and TGF-β1 expression, reduces Treg proportions in peripheral blood, and modulates the immunosuppressive microenvironment of gastric cancer. Our findings provide preliminary mechanistic insights and a basis for future investigation into using moxibustion as an adjunctive therapy alongside chemotherapy for gastric cancer through immune regulation. This study supports the rationale for further exploration of traditional Chinese medicine’s immune regulatory mechanisms in tumor treatment.

## Data Availability

The datasets presented in this study can be found in online repositories. The names of the repository/repositories and accession number(s) can be found below: The RNA-Seq raw count matrix and corresponding clinical information (STAR-Counts, GRCh38) of 33 solid tumors in TCGA (The Cancer Genome Atlas) were downloaded from GDC Data Portal (https://portal.gdc.cancer.gov), and the expression data of GTEx normal tissues (adjacent non-tumor tissues) were obtained through GTEx official website (https://gtexportal.org/home/datasets,V8 version).
